# Part II. General Review of Periodicals

**Published:** 1828-01-01

**Authors:** 


### Quarterly Periscope,

### PART II.

(GENERAL REVIEW OF PERIODICALS.)

### 1. Rheumatism—Spinitis.

M. de M. aged about 50 years, had enjoyed good health till his 48th year, with the exception of a gonorrhœa, followed by a stricture of the urethra. He had never been addicted to intemperance of any kind, though he was fond of good living and the enjoyments of convivial society. Eighteen months before the date of report, he became affected with an intermittent fever, which continued some months, in spite of every treatment, and then disappeared, leaving him rather debilitated.

A second time he was seized with intermittent fever, which resisted the usual means, and Dr. Potain was called in. The fever gave way to leeching the anus, and afterwards the administration of quinine and opium. At this time, he complained of the difficulty in making water, but did not take any remedial measures for that affection. In the Summer of 1825, this gentleman was again attacked by the intermittent, which soon gave way to the measures above-mentioned. Two months afterwards, he was suddenly seized with extreme dysuria. Leeches to the perineum—warm baths—lavements—diluents. The dysuria persisted—a catheter was tried to be introduced, but in vain, as the urethra was too irritable. By the soothing means above described, some water was made to pass, and, after a few days, a consultation was had, and the catheter again tried, but the stricture could not be passed. He now repaired to Paris, and was under the care of Dubois and Segalas. The latter applied the caustic bougie, and re-established the current of urine. The patient returned to St. Germain, (the place of his residence) where he was soon afterwards seized with rheumatic pains in the upper part of his back, shoulders, and arms. These pains were disregarded for a week, but then became so acute, that he could bear them no longer. Dr. P. visited him, and found him suffering severely, but without any fever. Thirty leeches were applied to the shoulders, succeeded by emollient cataplasms. He was put upon milk and vegetable diet. No relief followed these measures. Two vapour-baths were taken daily for five days, and although they were followed by profuse perspiration, they produced no mitigation of the pains. Every afternoon there was an exasperation of these pains, attended with fever, and preceded by some chilliness. These phenomena led to the quinine and opium once more, but they failed to arrest the disease. Twenty-five leeches were then applied every day for five days, followed by cupping-glasses over the bites. After the third day, the pains ceased entirely in the arms and shoulders, and were considerably diminished in the spine and neck. It was now discovered that, with the exception of the fingers, the upper extremities were paralyzed. Two or three days after this, there was experienced some difficulty in walking, and Dr. P. fearing that the paralysis would become general, applied a large blister to the spine, and demanded a consultation. The latter was held, and the physician called in, *openly, before the patient, blamed the measures that had been pursued,** and prescribed tonics and nourishing diet.

* It is hardly necessary to express our utter detestation of such a diabolical proceeding as the above. If the consulting physician was certain that the preceding measures were entirely wrong, or even

Dr. P. objected to the former, but acquiesced in the latter. This new consultant attributed the gentleman's complaint to "*un vice vénérien*," although he had not been in the way of any poison of this kind for ten or fifteen years! M. Potain thought, on the contrary, that there was some inflammation about the spinal cord, and that, consequently, the patient's life was in jeopardy. This opinion was not at all participated by his colleague, and the patient determined to put his trust in the latter. M. P. therefore, withdrew. Six days afterwards, M. P. was summoned to meet in consultation, to which were added M. Segalas and M. Dupont. The patient was found incapable of moving himself in bed. The pulse was quick, but the skin was not hot—evacuations could only be procured by injections—and the urine, which contained puriform matters, was obliged to be drawn off by the catheter. In fact, the poor patient was in a state of almost complete paralysis. Messrs. Segalas and Dupont coincided with M. Potain in opinion, and the *liberal consultant* above-mentioned could only be envied, we think, by a man marching solemnly to the new drop. All the measures proposed were resolutely resisted by the *liberal* doctor, and, in 24 more hours, the scene closed.

*Dissection.* At this disagreeable part of the business, the LIBERAL did not attend. M. Dupont, however, was witness to the dissection. The spinal column was laid open throughout. From the fifth cervical to the eleventh dorsal vertebra, the membranes of the spinal canal were seen to be intensely inflamed, and covered with a sanguinolent effusion. The membranes were also thickened. The spinal marrow itself was similarly inflamed, and for a similar space. It was also softened in consistence. The time occupied by this tedious dissection prevented an examination of the other parts of the body, as the priests now broke in, and immediately removed the corpse.—REVUE MEDICALE.

*Remarks.* Two of the members of the Royal Academy of Medicine were ordered to report upon this curious and melancholy case, and the said reporters have learnedly discussed the question, whether the spinal inflammation was owing to the repeated attacks of intermittent fever, or to the rheumatism. They decided that it was to the latter, this phlogosis of the medulla spinalis and its membranes was to be attributed. For our own parts, we do not see any decided proof that either of these maladies was the primary cause of the fatal inflammation above-mentioned; but, of the two, we should be inclined to take the intermittent as the most probable cause. This is the opinion of the original writer of the case, M. Potain. We were not a little surprised to find that no notice was taken by the Academy of the flagrant violation of medical etiquette and common ethics, committed by the second consultant in this case. It was an essential and important part of their duty to animadvert, in the strongest terms of reprehension, on his conduct. Indeed, it is a great pity that there is not some honorable tribunal to appeal to on such occasions. If our colleges and corporate bodies looked to any thing but their own private interests, they would take especial cognizance of all breaches of medical decorum and propriety in the classes of medical society over which they preside—or pretend to preside. But this seems to be no concern of the DIPLOMATIC Corps. A formal, and often an imperfect examination of the candidate, is all the *trouble* they take—the other part of the ceremony, the reception of the fee, is quite a *pleasure*. The PRESS, however, will probably rouse them from their halcyon slumbers—and the rising gale of public opinion may, perchance, so shake the fig-trees under which they placidly repose, as to disturb their—

"Golden visions and romantic dreams."

---

2. HYPOCHONDRIASIS CURED BY AGUE. The subject of this curious complaint was a gentleman, 55 years of age, meagre, nervous, and endowed with great sensibility of the digestive organs, (doué d'une vive sensibilité des organs digestifs,) which sensibility was less in cold, and greater

---

detrimental, he had no right to allow his sentiments to become known in any manner to the patient or friends, unless the first practitioner obstinately refused to hearken to his suggestions.

in warm weather. In the hot season of the year, he was teased with sense of distention in the stomach and bowels, loss of appetite, and tedious difficult digestion. As the Winter approached, these symptoms diminished. He was fond of hunting, and pursued this exercise both in Summer and Winter. He had been subject to the above symptoms for some years previous to the date of report; but they had considerably yielded to time and very trifling means, when, during the Lent of 1824, he determined to live in the most rigid forms of that religious fast, subsisting chiefly on fruit and vegetables. At this time, also, he met with some moral afflictions, and the consequence of both these causes was a state of sombre and morose melancholy. He shunned society—became possessed of the most triste ideas—passed his nights without sleep, or in frightful and troubled dreams. In this state he was, as usual, advised to cheer up—enter into society—and make himself happy—things easily said, but not so easily done! In short, this gentleman became completely hypochondriacal. In July, 1825, he consulted M. Bonnardiere, his *morale* being considerably improved, but the *physique* in a very depressed condition. The digestive functions were greatly impaired—he had pain and weakness in his ankles and wrists, with various other unaccountable ailings. M. B. now administered the cinchona in powder, by which medicine the appetite was restored, and the mind greatly relieved. He entered into society, and began to partake of his usual active exercises. In one of these, he over-exerted himself, and the whole of the dyspeptic symptoms returned, together with the hypochondriasis. On the first days of August, the patient had some febrile movements in the system, and became so weak that he could not walk without the aid of a stick. In fact, a marked intermittent fever was established, and our author, borrowing a hint from Hoffman, determined to let the fever take its course, for some time at least, with the hope that it might cure the hypochondriasis. During this experiment, there appeared some indications of mischief obtaining in the liver, as evinced by pain, tenderness, and some degree of fulness in that region—the strength was rapidly reduced by each paroxysm—and the Doctor, on the 17th of the above month, considered it unsafe to withhold any longer the quinine, which was accordingly administered. By the 24th, the period of the paroxysms was only marked by a slight cough and a loss of voice; on account of which it was not deemed necessary to interrupt the quinine. In the beginning of September the patient began to recover strength and spirits. He now took gradually increased exercise, and his appetite and digestion improved, and his amendment, corporeal and mental, was such as to surprise his friends as well as the doctor. Some months afterwards, he had a relapse of the intermittent, but not of the hypochondriasis, which soon gave way to the sulphate of quinine. He has since remained quite free from his hypochondriacal ailments, and enjoys perfect health.—*N. Bibliot. Med.*

The author cites several writers, who have maintained that fever has a powerful influence in checking chronic affections. Among others, he quotes Pugol and Dumas. There may be some reason to suspect that, in this case, the same cause which produced the intermittent may have previously operated in producing the affection of the stomach and the symptoms of hypochondriasis. The patient may have inhabited a situation where malaria abounded, and which shews its effects in various other ways besides common ague. We suspect that the hypochondriasis, in this case, was cured rather by the remedy administered for the ague, than by the ague itself. But of this we will leave others to judge, having presented them the naked facts of the case.

---

### 3. Gangrene of the Feet from obstructed Aorta.

*Case.* Count C———, aged 66 years, of athletic constitution, keen appetite, and a very hearty eater, had always kept himself in a state of plethora, with tendency to cerebral congestions and gastric irritation. From these affections he was usually relieved by sanguineous depletion, especially leechings; but his appetite being too powerful, he was never sufficiently guarded in his diet. In the Autumn of 1826, the Count became subject to an unpleasant sensation of heat in his feet, particularly when walking, which obliged him, after a promenade, to put his feet on cold marble to allay the heat. In the month of December, of the same year, the patient was seized with acute pain in one of his hams, which was removed by leeches. Next day, the pain settled on the tendo-achillis and neighbourhood, and appeared of a gouty nature, there being some swelling and redness. One evening, after a rather too free dinner, and a glass or two of strong wine, the pain became greatly exasperated, attended with quick pulse and hot skin. Leeches were applied without relief, and an attempt was made to bleed from the arm, but without success. The pain increased—delirium came on—with constant jactitation, and screaming. On examining the part, some vesications were seen, and soon afterwards gangrene manifested itself unequivocally. A troublesome hiccup now supervened, and could not be stopped. In the course of a few days the gangrene had invaded the whole of the leg—the patient became comatose—black spots appeared on the other extremity, and death closed the scene.

*Dissection*. The heart was found enveloped in a thick layer of fat, and intimately adherent to the pericardium throughout. The organ was flaccid, softened in structure, and its cavities dilated. The semilunar valves of the aorta were ossified, and the vessel itself considerably dilated, especially at its arch. About an inch and a half below the origin of the cœliac artery, the aorta became completely ossified, and its bore almost entirely obliterated. There were only two small passages for the transmission of blood, not more than half a line in diameter each. The iliac arteries were also interrupted, and nearly obliterated in several places. The femoral, or rather the inguinal arteries, about the crural arch, were rather dilated, thickened in their coats, and much ossified. Below the crural arch, the left femoral artery was merely thickened, whilst that of the other side was red, and filled with clots of different degrees of consistency. The vessels of the mortified parts participated in the condition of the surrounding soft tissues. The veins of the lower extremities were thickened, and obstructed. There were various marks of inflammation and thickening in the stomach and bowels, with considerable serous infiltration into the ventricles and between the membranes of the brain.—*Broussais' Journal.*

*Remarks*. It appears that the Count had been blind for several years, without any appreciable lesion of the optical apparatus. In the examination of the brain, it was found that the tubercula quadragemina and the optic nerves were not more than one half their natural size. The propensity of the Count to good eating and drinking has been observed. The state of the stomach accorded with this propensity. The mucous membrane was in a state of hypertrophy, and presented a remarkable contrast with the state of atrophy in which were found the optic nerves. The obstructions in the aorta, and in other parts of the arterial system, were owing, in M. Broussais' opinion, to a general inflammatory condition of the vascular system. The gangrene of the lower extremities was also, no doubt, the result of this obstruction to the regular supply of blood to those members.

---

### 4. Pericarditis and Hypertrophy of the Heart in a Child.

Dr. Menara has published an interesting case of this kind, shewing at how early a period the heart may become enlarged in children.

*Case*. A female infant had, from her birth till the age of five months, enjoyed apparent good health. Five of her brothers and sisters had died of cerebral affections. In the beginning of January, the little patient caught cold, which took on a more severe character than usual. On the *12th*, her breathing was oppressed, pulse full and hard, skin hot, cough frequent, but moist. She sucked with avidity, but often vomited. *13th*. Better in all respects; the chest sonorous throughout. *14th*. A strong exacerbation, preceded by a cold stage. The abdomen became distended and tender—cough, attended with oppression and râle. *Emollient drink—blister to the arm.* *15th*. Respiration more oppressed than ever. *Blister to the other arm.* The breathing was wheezy (ralante) in both lungs—and, on percussion, there were some dull spots beneath the scapulæ—pulse 140. These symptoms increased in intensity, and the child died on the 17*th*.

*Dissection.* The cranium was more ossified than usual at so early an age, the sutures being firm, and the anterior fontanelle closed. The brain was sound, but very voluminous, as was the spinal marrow. The lungs adherent by layers of albuminous concretion, with some serous effusion in the right side. The lower portions of lung were hepatized—the superior portions sound. The mucous membrane of the trachea and bronchia was rather thickened, and the tubes loaded with mucosities. The pericardium was found distended with gas, and also contained about two ounces of reddish serum. In several places, on the internal surface of the pericardium, reflected and non-reflected, there were layers of an albuminous secretion, some of them three or four lines in thickness. The pericardium itself was considerably thickened. The heart was one third larger than natural, this increase of size being produced by an inordinate thickness of the parietes of the organ.—*Tours Medical Journal.*

*Remarks.* The Editor of the Journal hazards some speculations on the connexion of the advanced ossification of the skull and development of the brain, in this child, with the congenital hypertrophy of the heart. It is not impossible that the too vigorous circulation, resulting from this congenital hypertrophy of the central organ, may have had its influence on the cerebral mass and its bony envelopes; but, we were not a little astonished to find the Editor pass over the subject of treatment, in this case, without a single observation. There cannot be a doubt that the child's life was lost for want of depletion. Not a drop of blood was drawn, although the symptoms of thoracic inflammation were as clear as the sun at noon-day, and required the most vigorous system of blood-letting, especially leeching. The congenital hypertrophy was not necessarily fatal; for many people have large hearts through life, without much inconvenience. We trust that the above example will prove a lesson to all practitioners, not to neglect examining the chests of children affected with cough, or other symptom of inflammatory action. The state of the thoracic organs can be very satisfactorily ascertained in children—much more so, indeed, than in grown people.

### 5. Epidemic of Groningen.

By Professor Bakker.

The City of Groningen suffered a dreadful visitation of epidemic fever in the years 1825 and 1826—especially in the latter year, when 2448 men and women fell victims to the reigning malady. Looking geographically at the soil and climate of Holland, intersected as it is by innumerable rivers and canals, and with such a large portion of its surface on a level with, or even below the level of the sea, one would be led to expect that the inhabitants, crowded as they are, would enjoy a much shorter range of existence than in other countries, as France and England for example. Yet, history proves to us, that the range of life is longer in Holland than in France—and that epidemics are comparatively rare. Thus, in France, it is found that, out of every 38 individuals, one will annually pay the debt of Nature, while in Holland, it is one in 42—in Belgium, one in 48—and in the province of Groningen, where the late mortality prevailed, it is only one in 49 and a half. This seems the more astonishing when we contemplate the many local causes which ordinarily give rise to fevers and other diseases throughout the world. The sources of miasmata must be more numerous in the low countries than in almost any part of the earth; but then, the *low temperature* of the atmosphere fortunately checks the evolution of these deleterious agents, and thus preserves the inhabitants from a terrible enemy, as nursed under brighter skies and more delightful climates. Every now and then, however, an inundation of the soil or an extraordinary range of atmospheric heat, calls the pestiferous miasms into full activity, and then, as at Walcheren or Groningen, the mortality is terrific.

During the Autumn of 1825, mild tertian and quartan fevers were seen in the City of Groningen, which continued to prevail during the Winter, and also in the Spring of 1826. But it was not till the month of June of the latter year, that the mortality became at all alarming. At this period, a diarrhœa, of a fatal nature, began to prevail, especially among children. By the middle of July, the epidemic became formidable, taking the gastric and bilious characters, accompanied by violent cephalalgia. It was clearly, however, of the *intermittent* type, though sometimes only remittent. The diarrhœa was, during one month, the prominent feature of the epidemic—but, about the middle of August, the tertian form of the fever was unequivocal, sometimes changing into the double tertian type—a change by no means advantageous to the patient. During the next two months, the malady raged with great intensity and fatality.

The disease, at this period of its acmé, commenced with a rigor of short duration and moderate in degree—slight acceleration of pulse—pains in the head, loins, and limbs, especially in the lower extremities. The tongue was furred, and the patient had bilious vomitings, sométimes during the cold fit—sometimes at the close of the paroxysm. These spontaneous vomitings were the means by which Nature got rid of large bilious secretions, but they were not indications for the administration of emetics or strong cathartics, which generally proved injurious. On the contrary, gentle excitants were more efficacious in removing the diarrhœa and gastric irritability. But the sulphate of quinine was still more serviceable. This specific almost always succeeded, provided it was given in the dose of fifteen to twenty-five grains in the course of the apyrexia. If, instead of this treatment, Nature was left to her own efforts, or the disease was attacked with sanguineous evacuations or drastic purgatives, the patient generally died apoplectic in the next paroxysm, or fell into a state of coma or dysentery, with delirium and prostration of strength, that ultimately proved mortal in the majority of cases. There were few, if any, instances, where the fever degenerated into the typhoid or putrid form, although thousands of unfortunate patients never had any medical assistance whatever, or only some trifling remedies unskilfully administered. In respect to crises, there were few opportunities of observing this operation of Nature, since the most strenuous exertions were made by the physicians to cut the disorder short by remedies, especially the sulphate of quinine. Still, they had occasion to observe a few instances where the fever seemed to terminate by critical sweats, diarrhœa, vomitings, or copious depositions in the urine. The disease was invariably of an asthenic character, and never inflammatory. This, they think, was proved by the symptoms, by the treatment—and by the termination. They do not add—by the *dissections*. But of this hereafter.

Relapses were very common—nay, almost inevitable. The reasons were two-fold—the protracted debility of the subjects, and a continuance of the original miasmatic causes. Dropsy was a very common sequence of the fever, and came on very suddenly. It was sometimes anasarcous—sometimes ascitic. The disease sometimes terminated in a singular eruption of chronic aphthæ on the tongue and throat, evincing their existence also in the œsophagus and stomach by acute pain in those parts, or a copious secretion of viscid mucus. There was, also, an occasional eruption on the surface of the body, in various parts. The debility of intellect, as well as of body, left by this fever, was sometimes so great, that the patient was entirely deprived of memory for a considerable time after the cessation of the fever.

*Appearances on Dissection.*

As the epidemic presented a great uniformity in its symptoms, so there was a corresponding uniformity in the *post-mortem* appearances. Dissections, however, were not so numerous as could be wished, owing to the extent of the mortality, and the inability of the physicians to attend to pathological investigations. Nevertheless, Dr. Hendricks, physician to the public hospital, opened 107 bodies, and communicated the results to the authors of the paper under review. A table is given of the different appearances on dissection; and we find, that the spleen suffered much more than any other part. Thus, there were 66 bodies presenting inflammation of this organ out of 107. Next to the spleen, the liver bore the onus of disease among the abdominal viscera. Thirty-two bodies shewed this species of lesion. In the whole number of dissections, (107) there were 26 cases where the digestive tube was inflamed, either internally or externally. Forty-two bodies shewed affection of the brain or its membranes, or serous effusion into the ventricles or between the meninges. Thus, each theorist had something to support his doctrine, but nothing to confirm it. Broussais and Clutterbuck would have found it difficult to maintain their exclusive hypotheses from these documents. There is certainly more to support the doctrine of fever being a general disease in the beginning, and taking a local determination in the course of the malady. Of the 107 patients above-mentioned, twenty exhibited, during life, symptoms of disturbance in the cerebral and nervous system—23 had dysentery—14 had diarrhœa—13 shewed signs of putridity—and 16 were dropsical before they died.

Nothing transpired during this epidemic to sanction the idea of its possessing any contagious character. Between 30 and 40 medical men (physicians) were busily employed, but none of them died. Some of them were taken ill, either from fatigue, or subjection to the same causes of disease with their patients.

In respect to the remote causes of the epidemic, they were ascribed, *first,* to an unusual increase of aqueous emanations —*2ndo,* to the high temperature of the Summer heat, which greatly augmented the said emanations—*3dly,* to the sudden vicissitudes of temperature—*4thly,* to the drying up of many marshy plains and pools, by which a great surface of mud, &c. was exposed, and a copious disengagement of miasmata effected.

From the above causes, resulted a bilious fever, early in the Spring, which soon changed into a pure intermittent, and afterwards into a remittent, with affection of various viscera. The same remedy (the sulphate of quinine) succeeded throughout all these changes and seasons—"and many hundreds of the citizens of Groningen would have been saved, had there been a sufficient supply of this invaluable febrifuge."

Groningen contained about 28,000 inhabitants previously to the epidemic, and during the months of August, September, and October, 1826, there were not less than 8,000 people affected with the fever —or more than a fourth part. Of these, 569 died in the above period, or about 1 in 14.

The city of Groningen presents large and beautiful streets, so that, at first sight, it might be considered a most healthy situation; nevertheless, it incloses within its walls many powerful causes of disease. One quarter of the city is too much crowded in its population, and there the epidemic raged severely. But this part is not inhabited by the lower orders—therefore the cause of sickness could not be attributed to want of cleanliness, good food, &c. But there was another cause, the immediate vicinity of the canal which leads to the sea, and from whose bottom and edges miasmata were doubtless disengaged, though not in any very large quantity. This city, however, is exposed to a still more powerful source of miasmata, namely the numerous cloacæ which are every where met with. These cloacæ are always filled with animal and vegetable matters in a state of putrefaction, and, in the hot weather, disengage an immense quantity of stinking effluvia, which contain, without doubt, the seeds of fevers and other diseases. This terrible epidemic will probably induce the local authorities of the place to pay more attention to the state of drains, canals, and common sewers, in order to guard against another visitation of so terrible a scourge. —Journ. Gén. de Médécine, *April,* 1827.

---

### 6. Gastro-Cerebral Inflammation, with some (supposed) symptoms of Hydrophobia.

In a late Number of M. Broussais' Journal, we have the case of a boy five years of age, which presents some characters of interest. This boy, of vigorous, but irritable constitution, was seized, on the 1st of May, with symptoms of gastro-enteritis, which were removed by leeches to the epigastrium and diluent drinks. In the night of the 12th May, he awoke from his sleep with a loud laugh, which was quickly followed by piercing cries, and then convulsive movements of the whole body. His face was flushed, eyes sparkling—and he attempted to bite, and to squeeze tightly in his arms, every thing that came in his way. The parents were alarmed at hydrophobia, and summoned two physicians. When they arrived, the patient was in an interval of calm—lying without sense—his eyelids shut, but the balls rolling about underneath—pupils greatly dilated, but still sensible to light—face of a purplish cast—skin burning hot—pulse contracted, hard, and quick—carotids beating strongly. Twelve leeches were applied to the regions of the jugulars—sinapisms to the feet—cold lotions to the head. In five hours the physicians returned, and the scene was greatly altered. The little patient seemed in the jaws of death. His whole surface was pale and cold—pulse imperceptible—the respiratory movements were the only indications of life. These phenomena resulted from an excessive hæmorrhage. The leech-bites were stopped—stimulating frictions were applied, and some spoonfuls of soup were injected into the stomach. The boy gradually recruited, and a corresponding reaction took place, when diluents were prescribed. During the succeeding day and night he had repeated vacillations between paroxysms of excitement and alarming states of collapse. The latter, however, were the more predominant; but, at the end of the third day, he had become sensible—his appetite gradually re-appeared, and by the 22d May, the boy was convalescent, and the physicians took their leave.

*Remarks.* M. Broussais observes that the above is a good example of the energetic effects of local bleeding carried *to syncope*, for the cure of acute inflammation in young subjects. Cerebral inflammations are the most rebellious of all, in this class of patients, and the Professor doubts whether this boy's life would have been saved, had the hæmorrhage from the leech-bites not gone on unintentionally to syncope. Notwithstanding the authority against us, on this point, we much doubt whether the above patient was actually affected with acute inflammation of the brain and stomach, as he is stated to have been. The manner in which he was seized—the violent vacillations of the phenomena, from intense excitement to frightful collapse, three or four times in the 24 hours, are not the phenomena which we have observed in acute inflammations of either brain or stomach. The complaint, in our humble opinion, was some powerful irritation of the gastric or intestinal nerves, affecting sympathetically the whole nervous system, and thus imitating inflammatory action, when nervous irritation was at the bottom of the whole. Many are the examples which we have seen of this deception. Neither do we observe any phenomena in this case, which can authorise either M. Broussais or the physicians who attended the patient, to say that there were "*quelques symptômes de rage*" manifested by the boy. If attempts *to bite* be indications of hydrophobia, there is many a female affected with that disease in common paroxysms of hysteria. We wonder that such a man as M. Broussais should permit the above observation in this statement of the case, as compiled by the attendant physicians.—*Annales de Med. Phys.*

---

### 7. Spontaneous Paraplegia.

Mr. Iliffe lately read a case at the Medical Society, Bolt Court, which has been reported in the Lancet, and headed "Spontaneous Paraplegia." We know not why the term "spontaneous" should be applied to a disease of the spinal marrow causing paraplegia, any more than to a disease of the brain causing total or partial paralysis. Be this as it may, the case gave rise to a hot discussion in the Society. The following are the principal features of the case.

A gentleman, ætat. 25, became indisposed in the Summer of 1827, complaining of "a gradual diminution of strength," with pains about the wrists and ankles, and towards the end of August, a slight difficulty in making water. On the 2d September he dined in Fleet Street, and in the evening was sick. The difficulty of making water increased, as did the weakness of the lower extremities. Mr. I. saw him, and ordered some medicines. On the 4th, he had nearly lost the power of the inferior extremities—pain in his loins—water dribbling from him—no general febrile symptoms. *5th.* "Has had considerable uneasiness, during the night, along his back, and under each scapula,"—no power of the bladder. Mr. Callaway called in consultation—catheterism—blister to the spine—ammonia and cascarilla—Plummer's pill. *7th.* Little alteration *8th.* Pulse 80—no pain—appetite good—

spirits depressed—still no power in the limbs. *9th.* Passed a restless night, and is rambling to-day—thirst—hot skin—tongue dry—bowels torpid. *11th.* Dr. Back called in. Calomel and cathartic extract—nourishing diet—blister to be healed. *12th.* Pulse 120—quinine—calomel and colocynth—in the evening, the pulse 164—abdomen tense. Died on the 13th September.

*Dissection.* This was made in a very able manner by Dr. Hodgkin. We shall pass over many of the minutes of dissection, as not bearing on the immediate seat of disease. The arachnoid was partially opake and remarkably firm—considerable effusion beneath the arachnoid—pia mater injected—substance of the brain natural. The dura matral covering of the spinal medulla was sound—the pia mater considerably injected—in two spots, of an inch or more in length each, the pia mater was black—the medulla appeared to have experienced "considerable pressure from its pia matral envelope." The consistency of the medulla was, throughout, softer than usual, and almost the whole of the dorsal portion was "much softer." Opposite the black patches above-mentioned, the medullary matter "*contained numerous minute dark-coloured ecchymosed points.*" In one place the medulla was so broken down as to produce "the appearance of a cavity." "Some spots were nearly black." This state existed, in a greater or less degree, throughout the dorsal region. "The consistence of the most softened portions was similar to that of cream." The mucous membrane of the bladder was of a livid colour.

In the discussion which ensued, it was maintained by Dr. Johnson that the above appearances were the legitimate products of chronic inflammation, and it was as resolutely maintained by Mr. Callaway that neither the symptoms nor the appearances on dissection evinced the slightest connexion with an inflammatory process. It is not for us to pronounce on this point, and we leave it to the public to decide between Mr. Callaway and Dr. Johnson. We think there can be little doubt that the disease (by whatever name we may call it) had been going on for a considerable time before either Mr. Iliff or Mr. Callaway saw the patient—and, consequently, that it was far beyond the power of art when these gentlemen were consulted. The question, therefore, is principally pathological—and we would refer Mr. Callaway to the writings of Ollivier, Abercrombie, and many others, on this point. If Mr. C. will turn to the first volume of this Series, page 39, he will see a case quoted by Dr. Abercrombie from Professor Brera, which bears a very close analogy to the one in question.

P. S. In a conversation with Dr. Hodgkin, who made the dissection in this case, and, than whom, there is not a better pathological anatomist in London, that gentleman expressed his entire conviction that the appearances in the spinal marrow of the above patient were the legitimate products of inflammation.

---

### 8. Pneumo-phthisis Cyanotica.

In the 4th number or volume of our respected cotemporary, the Journal de Progres, there is a short article under the above head, extracted from Hufeland's Journal for February, 1827. This terrible affection (pulmonary phthisis with the blue disease) has engaged the attention of Professor Schonlein, who has described it minutely. It is generally developed at the age of puberty, and is rapid in its course, rarely continuing more than three months, and is remarkable for the affection of the venous system, the pulsations of the heart and arteries, (which are not always synchronous with each other)—by the blue colour of the face and lips—the narrowness of the chest—the meagreness of the extremities, except at the tips of the fingers which are generally swollen, and the great incurvation of the nails. To these symptoms succeed cough, pain in one or both sides, oppression about the chest, expectoration, at first sanguinolent, and afterwards purulent, accompanied by hectic fever in the last period of the disease. It differs from common pulmonary phthisis by the rapidity of its course, by the want of general emaciation, the dryness of the skin, the paucity of expectoration, the constipation of the bowels, and the cerebral phenomena.

The effective cause of the malady is *non-obliteration of the foramen ovale,* which experience has shewn to be more frequent in the female than in the male. It is thought that it is sometimes owing to hereditary disposition, as it is found that the children of parents who have organic diseases of the heart, are apt to be similarly affected. There are some other predisposing causes enumerated, but we do not deem it necessary to notice them.

On dissection of subjects who die of this disease, we generally find the venous system of the brain gorged with blood, the ventricles distended with serum, and *one or both phrenic nerves swollen and indurated, or else in a state of atrophy.* In the heart, the foramen ovale is open. The blood throughout the whole system is found quite liquid—the lungs partially ulcerated—the liver enlarged.

The cure of the disease is beyond the reach of art, since we cannot remedy the malformation of the heart, on which it mainly depends. But the author thinks that something might be done by getting the individual past the period at which the disease usually terminates fatally. This is to be done by the greatest repose —by avoiding aliment of a hydrogenated quality—by living chiefly on vegetables and water, &c. And as the oxygenation of the blood in the lungs is very imperfectly performed, this defect is to be remedied as much as possible by soliciting the action of the other secretory organs with which the lungs are in accordance, as the kidneys, liver, skin, &c. At the same time, the respiratory organs are to be guarded by the greatest attention to a regulated temperature, and defending them from catarrhal affections. We shall now give some particulars of a case in illustration of this formidable disease.

*Case.* A. Gotlieb, aged 16 years, was said to have enjoyed good health till the year preceding, when he became affected with pains in the left side of the chest, dry cough, dyspnœa, and fever. The village barber was called in, who practised a large bleeding from the arm. This measure diminished the pain in the side; but the other symptoms continued the same. On the 28th January, the tenth day of the illness, the author was called in. He soon discovered the radical defect in the central organ of the circulation—as evinced by the usual symptoms of cyanose, described above. The pain in the side had now returned, and the difficulty of breathing amounted almost to orthopnœa. The fever was slow, the expectoration mucous, the appetite gone. Quietude, aqueous drink, and vegetable diet, were prescribed; while digitalis, nitre, hyosciamus, &c. were ordered. In a few days, the oppression and pain were diminished, but the expectoration had become sanguinolent, and the fever was increased every evening. Eight grains of calomel were given night and morning, in order to procure stools. *3d. Feb.* The expectoration is now pure blood; but the cough and oppression are somewhat relieved. Acids were given, and a tepid muriatic acid bath ordered once in 48 hours. By these means the hæmoptysis was checked; but purulent expectoration succeeded. On the 26th March, there was another discharge of blood from the lungs, and life was threatened. Venesection and acids again stopped the hæmoptysis; and again the purulent expectoration became copious. The disease now made rapid progress, and the patient was carried off early in May, having evinced, for some time, considerable disturbance of the cerebral system.

*Dissection.* The venous system of the brain was gorged with blood—some serous fluid was found in the ventricles. The lungs were gorged in several places with blood, especially the upper part of the left lung, where there were several recent abscesses. The phrenic nerves were enlarged and indurated—and the left par vagum reduced to a kind of bouillie. The pericardium contained serum—the foramen ovale was open—the two auricles were prodigiously enlarged, but all other parts of the heart were natural. The liver was enormously enlarged, and quite lacerable.—*Journ. de Progrès, No.* 4.

---

### 9. Lithotomy.

Two cases are reported from Panton Square, the first of them fatal.

*Case.* H. Morgan, æt. 10, had been labouring under the symptoms of stone in the bladder for five years. When admitted, his sufferings were exceedingly severe, but the general health was good, although he was subject to febrile disturbance of the system. Having sounded, &c. Mr. Wardrop, assisted by Mr. Lawrence, proceeded to the operation, which was performed with a common staff, having a deep groove, and a large scalpel with straight and rounded back. The incision of the prostate not being sufficiently free, it was necessary to introduce the knife twice to enlarge it, when a stone, weighing above an ounce and a half, was extracted. When put to bed, he had a severe rigor and much pain, for which he took a large opiate. *Vespere.* Very low—pulse 100, small, and fluttering—pain in the abdomen and wound, through which the urine passed freely. *Another large opiate.* In the morning, the countenance was sunken and anxious—the pulse 140, but weak and fluttering—much internal pain, with tenderness of the abdomen. A large opiate was again given, with camphor and ammonia, but the patient continued to sink, and expired next morning.

*Dissection.* The bladder was greatly contracted, and the incision into it remarkably large. The peritoneum, generally, was vascular, particularly over the fundus of the bladder, where there was recent coagulable lymph, exactly resembling thick pus. The left kidney bore very decided marks of ancient inflammation.—Lancet, 208.

*Remarks.* It is evident that this patient sunk, not so much under the local inflammation, as in consequence of the shock given to the system generally by the operation. These cases, as Mr. Wardrop justly observed, are exceedingly difficult to treat—you dare not deplete for fear of the exhaustion—you are afraid to stimulate because of the inflammation. But might not the local action have been moderated here by local means, leeches, fomentations, &c. whilst, at the same time, the system was supported by the camphor and ammonia? We think so, though we are quite ready to grant that the odds were fearfully against recovery, whatever treatment was employed. The reporter, makes a very silly remark. He observes, that he was particularly struck, in this case, with the facility with which the largest stone may be extracted by the *scalpel* operation, *after the incisions have been made sufficiently free!* This is rather unfortunate, considering that, in this very case, they were obliged to employ repeated introductions of the scalpel, before the incisions actually *were* "sufficiently free."

---

10. On Extirpation of the Uterus.*

By Dr. Gustavus Hesse.

This operation, though one of the most formidable in surgery, has been practised for centuries past—and more frequently by ignorant or audacious Charlatans, than by regular surgeons. A German surgeon, however, (M. Osiander) rescued this operation from the hands of quacks, but, unhappily, he never could be prevailed on to give the details of his practice to the world at large. He simply stated, from time to time, that he had performed the operation, and promised a book on the subject; but he died at an advanced age, and has left no records of his cases—at least, that are available by the profession.

Osiander's operation was seldom imitated till of late years, when several cases have been published of partial and even of total ablation of the womb. It would be wrong to form too sanguine hopes from this operation, especially in cases of cancer uteri; but the experience of Osiander enabled him to aver, that extirpation of scirrhous uterus was as successful as extirpation of that disease in other parts of the body. All of his patients recovered from the immediate effects of the operation, though some of them relapsed afterwards, and died of the renewed malady.

This operation (extirpation of the uterus) is said to have been performed in a great many instances formerly. Baukin relates nineteen cases; and Schenk a far greater number. But it is justly doubted, whether many of these were not partial amputations of the organ, or removals of polypi, which had been mistakèn for enlargements of the womb. Baudelocque, however, considers it as incontestible, that the extirpation of the uterus had been successfully executed before his time. The operation has been performed

* Allgemane Medezin. Annalen. May, 1826.

too, when it was not designed—and the uterus has been mistaken for a polypus or other morbid growth, and extracted under this false supposition. In our own country, Clarke and Johnson have afforded examples of this nature; while Petit, Paletta, and others, have recorded instances on the Continent. From this it will be evident, that it is not very easy to get at the exact truth, in the relation of cases, even where there is not the smallest disposition to deceive, on the part of the operator.

Our author investigates the history and utility of this operation under three points of view—in prolapsus uteri—inversio uteri—and cancer of the organ.

1. *Prolapsus Uteri.* This appears to have been the case to which the operation in question was first directed, if we fairly interpret the writings of Ætius and Paulus Egineta. No doubt this measure was not resorted to, except in extreme cases—probably where the uterus was threatened with gangrene. In the case related by Hosack, (Med. and Philos. Register), the womb, at first prolapsed, had afterwards become inverted, and had degenerated into a scirrhous structure. The uterus extirpated by Ruysch, was in a similar condition; and that operated on by Wolff, was prolapsed and carcinomatous. Fodéré (Journ. Complement. Tom. XXI.) details a case of prolapsed uterus, where the organ was first tied with a ligature, and, two hours afterwards, cut away. The organ was changed in structure.

The ligature first, and resection afterwards, was the most general measure which was resorted to, both by Ancients and Moderns, but not the only means. Ambrose Paré relates a case where the knife was used without any ligature, and the woman recovered from the operation, but died three months afterwards of pleurisy. Another case is on record, where a bold and ignorant empiric cut away a prolapsed and inverted uterus, and, with it, several feet of intestine which had descended in the organ! Van Heer witnessed this exploit. We need hardly say that almost immediate death was the result. Langenbeck extirpated, with the knife alone, a carcinomatous prolapsed womb. After having divided, by a scalpel, the connexions of the vagina with the uterus, and dissected the peritoneum back off the organ, he removed the uterus, but encountered a tremendous hæmorrhage, from which, however, the patient recovered completely.

The ligature alone, without the knife, appears to have been seldom trusted to in this operation. Blasius mentions a case where a woman died three days after the application of a ligature. Dr. Marschall, of Strasburg, (in 1794) applied a ligature to a prolapsed and scirrhous uterus, but was obliged to cut it away on the second day, in consequence of pains in the abdomen and convulsions. He then had recourse to the knife, and the woman survived the operation ten years. Ruysch has published an unfortunate case where the ligature included the urethra of the patient, and death ensued.

2. *Inversion of the Uterus.* This state of the organ gave occasion to the operation in question, full as often as prolapsus uteri. There must, always, indeed, have been more or less of prolapsus accompanying inversion of the organ—and the Ancients did not make any very nice distinction between these two states. The inverted uterus has been, sometimes, cut away at once—sometimes tied with ligature, and then amputated—sometimes treated with the ligature alone. It has been totally, and it has been partially removed. Wrisberg relates a case where a midwife inverted the uterus, and then cut it away, thinking it was the afterbirth! The woman survived after a frightful hæmorrhage. This is encouragement for Sir Anthony and his ultra-delicate partisans. M. A. Ulmus gives the history of a similar exploit peformed by a midwife; but the result was immediately fatal. A third instance, in all respects the same, and with a similar catastrophe, is put on record by Fabricius Hildanus. In the *Annal. de Literat. Med. Etrang. à Gand. T.* XV. there is a case where a midwife, in dragging at the umbilical cord, inverted the uterus. The SAGE FEMME completed the job by cutting all away with a razor. The patient fortunately recovered by the application of ice, which stopped the hæmorrhage. These cases induce to the belief that, in the hands of a skilful surgeon, the danger of the operation is not so great as one would be led to suppose. Among the numerous cases on record, where the inverted uterus was extirpated, we shall only cite a few.

Vieussens and other surgeons were consulted in the case of a woman, where a tumour presented, the exact nature of which they could not ascertain. They all agreed that it should be removed, which was done, by ligature and the knife. It was then discovered that they had extirpated the uterus. The woman recovered perfectly, and lived fifteen years afterwards. The body was examined in the presence of several physicians and surgeons—and the fact was proved beyond a doubt. Mr. Baxter relates a case where the uterus was inverted, and, five weeks afterwards, the ligature was applied and the organ cut away. The woman recovered. A case is related in the Medico-chirurgical Transactions by Sir Astley Cooper, in which Mr. Windsor tied and extirpated an inverted uterus with success.*

The ligature *alone* has been used in but a few cases comparatively. Mr. Newnham's case is well known, and is fully detailed by him in an essay on the subject. M. Faivre (*Journ. de Medecine, Aout*, 1786) performed this operation by the ligature. The uterus had been inverted by the violence of the midwife, and was sphacelating. He applied the ligature. The patient was harrassed with vomiting, convulsions, and diarrhœa, till the 27th day, when the parts separated, and the patient, from that time, did well. Under the same head, may be ranged a case in the third volume of the Dublin Hospital Reports, communicated by Dr. Charles Johnson. An inverted uterus was mistaken for a polypus, and a ligature applied. The mistake was discovered, but it was deemed prudent to continue the ligature. In three weeks the tumour came away, and the patient did well. It appears that Dr. Johnson was in error, in his remarks on this case, by attributing to M. Petit the honour of having first applied the ligature for the removal of inverted uterus. German research has proved that this operation was performed by Rousset and many others, long before M. Petit existed.

* Vol. x. p. 356.

3. *Extirpation of Carcinomatous Uterus.* We are now arrived at the period when the operation of extirpating, partially or totally, the uterus or its neck, affected with scirrhus or cancer, (but neither prolapsed nor inverted,) has created a great sensation in the medical world. In this historical sketch, however, we must sometimes revert to cases where the above-mentioned conditions (prolapsus and inversion) accompanied the carcinomatous state.

The merit of first operating on the uterus in this dreadful disease is generally given to Osiander, though it is certain that Wrisberg *proposed* the operation long previously. But, as there is one glory of the sun, and another of the stars —so there is one kind of honour in *proposing*, and another in *executing* a hazardous and new operation. It is certainly very probable that, among the numerous instances of extirpation of the uterus, for inversion and prolapsus, there were some cases of scirrhus or cancer; but still Osiander has the merit of first amputating the uterus in a carcinomatous state, uncomplicated with inversion or prolapsus.

Osiander was greatly averse to total ablation of the organ. He dreaded the descent of the intestines, the hæmorrhage, &c. He, therefore, contented himself with the removal of the diseased portion of the womb, by means of scalpel or scissors. At first, he recommended and practised the drawing down of the uterus by means of a ligature, before cutting away the parts; but, he afterwards abandoned this measure, and trusted to the fingers introduced into the cavity of the womb, and serving as a guide to the cutting instruments.

Osiander performed this operation 23 times in 15 years, viz. between 1801 and 1816, yet he never published a single case in detail; but only gave a few particulars of the first operation to the public. Our knowledge of the facts of these operations is authenticated by those who witnessed them; but the original operator carried with him to his grave all the knowledge he possessed on the subject. This is a great sin in any man. Medical knowledge is derived from the public, as rain is derived from the ocean. The former should return to its *source* like the latter.

Professor Rust (*Med. Chir. Zeit.* 1813)

was the first, in Germany, to imitate Osiander. He extirpated the cervix uteri, according to the plan of Osiander, in the case of a woman, 50 years of age, who had a cancerous excrescence of this part, the size of a man's fist, the rest of the organ appearing to be sound. The hæmorrhage was very considerable, and was, with great difficulty, restrained. The patient died eight days after the operation, and, on dissection, the remainder of the uterus was found in a state of sphacelus.

Paletta *(Med. Chir. Zeit.)* performed the operation of Osiander, as he thought; but, on examination, the *whole* of the womb was found to have been removed, in a prolapsed and degenerated state. The woman died three days after the operation, with symptoms of peritonitis.

Graefe was the next who performed ablation of the cervix uteri, affected with a cancerous disease. He removed the parts with scissors. The hæmorrhage was arrested by the introduction of sponges soaked in cold water. Inflammation of the remaining portion of womb, of the bladder, and of the intestine, supervened, and reduced the patient to a state of great danger, but she ultimately recovered.

In France, the operation has been several times performed by Dupuytren. Previous to 1815, *(Bibliotheq. Med. Fevrier,* 1815,*)* this eminent surgeon had operated seven times. In one case the disease returned at the end of two years—in a second, there was a recurrence of the malady in 18 months. In a third case the woman was living and well in 1815, four years after the operation.

Bayle strongly recommended, and indeed brought into vogue, in France, the treatment of cancerous uterus by means of caustic. That distinguished physician had observed, in numerous dissections, that the change of structure under uterine ulcerations goes to a very limited depth—only two or three lines. The arsenical paste, and even the actual cautery, have, therefore, been frequently applied with success in France.

Numerous objections have been made by Wenzel, Zang, Siebold, and others, against Osiander's operation of *partial* ablation of the uterus. The main objection consists in the fact that, in such cases, there is *generally* disease, or strong disposition to disease, in the whole of the organ, and, consequently, there is great chance of the disease continuing or returning. It must be confessed that this objection very often lies; but the same may be urged against the operation in any or every other part of the body. There may be cases and circumstances to which this partial amputation of the uterus is applicable.

As to the question respecting the best method of operating, it is doubtful whether the ligature, the knife, or both in sequence, should have the preference. The knife would certainly be the best instrument, could we be sure of commanding the subsequent hæmorrhage.

*Total Ablation of the Womb.* Before this operation was attempted, Wenzel, Zang, and others, pronounced it to be impracticable—or, if practicable, that it would be inevitably fatal. Experience has shown how dangerous it is to prognosticate with confidence on the event of any surgical operation. Sauter was the first to disregard these croaking predictions, and try the experiment. The patient on whom he first operated was 50 years of age, and affected with a true cancer of the whole uterus. The operator first tried to draw down the organ with his finger; but, finding that impossible, he passed the fore and middle fingers of the left hand between the pubes and the uterus, and with these as a guide, he separated the connexions between the vagina and uterus, anterior and lateral. This done, he was enabled to draw down the uterus by means of a hook. A further separation of the organ from its neighbouring parts was effected, but not without wounding the bladder. It was now found impossible to remove the uterus without cutting through the peritoneum. This was done, and the cavity of the abdomen was actually laid open. The fingers were then passed up over the fundus uteri, and the attempt made to invert the organ forwards; in which attempt a quantity of intestine was protruded. This was returned—the antero-version of the uterus effected—and the organ finally cut away. Means were then used to prevent the prolapsus of the intestines, and, in two months, the wound might be considered as healed, with the exception of a vesical fistula. Four months afterwards, however, the patient fell a victim to a disease of the lungs. On dissection, there were found only some trifling adhesions between the ileum and the peritoneum, the result of the operation; the cause of death being in the lungs.—*Sauter's Work.*

*First Operation of Siebold.* This case is detailed in the 3d volume of the present Series, p. 264—6. It was fatal; but the mass of disease in the abdomen rendered it hopeless from the beginning—and, indeed, an improper case for the operation at all.

*Operation of Hoelscher.* This case is also given in the same volume immediately after the preceding one. It was fatal.

*Operation of Professor Langenbeck.* Two females were operated on by this gentleman.

*Case.* A woman, 30 years of age, was received into the clinical wards of Langenbeck, in Gottingen, on the 4th of January, 1825. She had had eleven children, and enjoyed good health till within three or four months of the date abovementioned. When examined in the hospital, she presented the following symptoms: viz. abdomen painful, especially on the right side—a great deal of fetid discharge from the vagina—two scirrhous tumours in the vagina preventing easy access to the uterus—neck of the womb ulcerated—internal parietes of the organ, as far as could be felt, studded with tumours—in short, the uterus was pronounced to be, not merely scirrhous, but affected with open cancer. On the 11th of January, Langenbeck performed the operation, above the pubes. An incision being made from the symphysis pubis to within two inches of the umbilicus, the parietes of the abdomen were cut through to that extent. The intestines and bladder being kept out of the way by assistants, the operator seized the uterus with his left hand, and, introducing a long pair of scissors shut, into the abdomen, he first separated the right ovary from the uterus, and then went on detaching the organ itself, together with the scirrhous tumour above-mentioned, till the whole were removed, the operation not lasting more than seven minutes. There was very little hæmorrhage, and no protrusion of the intestines through the vagina. The patient died 32 hours after the operation. On dissection, there were seen marks of extensive peritoneal inflammation, and a large quantity of coagulated blood in the pelvis. The bladder was gangrenous.

*Second Operation of Siebold.* A female, 30 years of age, of delicate constitution, (whose mother had died of cancer of the womb, at the age of 45,) had always enjoyed good health, and had borne some children. After her fifth accouchement, she entered the hospital of Berlin, in 1824, complaining of violent pains in the region of the uterus, of a darting and burning nature, extending often to the abdomen, and causing faintness. There was also a sense of weight in the hypogastrium, with frequent and painful micturition, bad state of the bowels, nocturnal sweats, shiverings and flushings alternately. There was a discharge from the vagina, sometimes inodorous, sometimes fetid, sometimes mixed with clots of blood. The projecting portion of the os tincæ was hard and very painful to the touch. The fundus uteri was also very painful, and turned a little backwards. There were various hæmorrhoidal excrescences about the anus. The conclusion was, that the uterus generally was affected with scirrhus. The patient ardently wished for an operation, and it was performed on the 25th July, 1825. The vagina was slit on one side, and the uterus being hooked with a needle and strong ligature, was removed by means of long scissors. The operation lasted 25 minutes, the patient supporting it with the most heroic fortitude. The hæmorrhage, at first, amounted only to a few ounces; but, twelve hours after the operation, there issued from the vagina ten or twelve ounces of pure blood. The patient sank the next day. Extensive inflammation was found in the abdomen.

*Second Operation of Langenbeck.* A female servant, 28 years of age, was received into the clinical wards of Gottingen Hospital, on the 28th July, 1825. She had borne her first child at the age of 19, and a second at the age of 24 years. This last accouchement was very laborious, and instrumental aid was necessary; but she recovered well, and continued so till 1824, when she began to experience lancinating pains in the pelvis, from time to time, especially in the left side. On examination, the abdomen was found painful on pressure; the os tincæ was projecting and hard to the touch; the uterus itself appeared sound on the right side; but, in the left, it presented scirrhous eminences, and was adherent to the vagina. From this part there was a thin sanious discharge. The finger introduced into the rectum felt the uterus hard and studded with scirrhous tubercles. The disease was, therefore, pronounced to be scirrhus uteri.

The operation was performed on the 5th August, at 8 o'clock in the morning, after proper preparation of the patient. The bladder being emptied, the perineum was incised backwards by a bistoury, in order to give more space to the operator. The surgeon took the hysterotome of Osiander, and commenced his incision in the right side of the vagina—then introducing the fingers of the left hand along the posterior paries of the vagina, between the rectum and uterus, he divided the peritoneum with the hysterotome, and enlarged the wound towards the bladder by means of the scissors. He then seized the fundus uteri with his fingers, (keeping the back of his hand towards the abdomen to prevent the descent of the intestines,) and drew it downwards, dividing the connexions, and removing the organ. The operation lasted 15 minutes. A sponge dipped in vinegar and water was introduced into the wound, and the patient put to bed. She died on the 7th, after midnight. The peritoneum was every where found coated with coagulable lymph, agglutinating the intestines together. The bladder was black—much blood in the pelvis.

Upwards of two years ago, (July 1825,) when we stated the case of Professor Siebold, we took the liberty of differing from our senior cotemporary of the North, on this point of surgery, and made the following remark. "We consider the extirpation of a uterus not previously protruded or inverted, one of the most cruel and unfeasible operations that ever was projected or executed by the head or hand of man." We still think so; and we believe we are borne out in this opinion by the results. Let us hear what Dr. Hesse says on this occasion. We shall quote from the French. "Dirons-nous avec M. Siebold:—*felix quem faciunt aliena pericula cautum!* Les resultats de ces essais ont été en effet tellement fâcheux, que tout operateur trop entreprenant, devrait, ce semble, s'arreter, au souvenir de ces monumens effrayans d'un zèle poussé à l'exces."

At the same time Dr. Hesse thinks that the result of Sauter's operation prevents the door from being completely shut against total ablation of the uterus, where there is neither procidence or prolapse of the organ. All we can say is, that we conceive the *total* extirpation of the womb, and consequently the opening into the cavity of the abdomen at the part, is an operation not justified by any thing yet put upon record. The partial extirpation of the organ is quite another thing, because it does not expose the peritoneum to incision.

---

### 11. Diseases of the Nail.

What a magnificently clever gentleman it is who reports from Panton Square! Why, really, if there were not another paper in the Lancet, his observations alone would be well worth the eightpence. A little while ago he set the profession to rights about the diseases of their knees, and now he has kindly consented to look to their toes. It appears that Mr. Durlachre, "the celebrated Royal Chiropedist," has lately been showing his mode of treating affections of the nail in the Hospital of Surgery, and this being an opportunity that our Matty Marvellous could by no means let slip, he has filled three mortal columns with the praise and glory of himself and Mr. Durlachre. As the method of operating seems to be really deserving of attention, we shall place it before our readers, leaving all the flowers of rhetoric, &c. behind. The principle "consists in cutting the former (the nail) through, without injuring that portion of the cuticle, which is reflected over the singularly sensible surface which lies below." The detail of a case will best explain the process.

*Case.* ———, æt. 20. The soft parts around the nail of each great toe are red, swollen, and excessively painful; the skin at the edge of the nail has ulcerated, and a bleeding fungus has arisen. He has undergone several operations on the part without success, and each side of the nail being affected here, the operation was more complicated than usual. The root of the nail being cleaned, and the distances marked, Mr. D. with a very small fine knife, resembling Hey's cataract needle, made cautious incisions through the nail, until he had reached the epidermis below, occasionally using a small lever, to show how far he had penetrated. The nail was then gently severed from its attachments, by means of a small pair of forceps, having a rounded point with a sliding clasp. There was no pain nor bleeding, and the relief given was immediate. We certainly think this method of Mr. Durlachre's preferable to the ordinary one of introducing the blade of scissors between the nail and "quick," and so slitting it up. The pain is really excruciating, and besides, it is frequently no easy matter to cut the nail, however you may have paired and thinned it beforehand. With regard to escharotics, we are convinced that, unless the disease be quite in embryo, they will not do *alone*. —*Lancet*, 209.

---

12. Fracture of the Spine—Extension employed.

J. Harlow, æt. 19, was brought into Guy's, July 16th, with injury of the spine, in consequence of a quantity of gravel falling in upon him whilst "excavating." Examined by Mr. Key, there was found depression of the spinous processes of the last dorsal vertebræ, whilst those of the upper lumbar apparently projected, in addition to which there was some lateral distortion. The lower extremities were quite paralysed, and the integuments of the abdomen devoid of sensation. Mr. Key determined to try and *reduce* the displacement of the vertebræ; accordingly, pillows being placed beneath the belly of the patient, the back was bent over them, but without effect; the man was then placed on his side, with his body bent, a napkin passed under the arms, and one above the ilia, and extension in either direction made by assistants, until the bones resumed almost their natural situation, *with a distinctly audible noise*. Mr. Key thought the patient was better, but the amelioration was trivial. *Cuc. cruent. ad* ℥vj.—*house-physic*. 17*th*. Much the same; lower extremities and bladder paralysed—little pain. *Almond mixture, with* 15 *grains of the nitrate of potash, thrice a day. Twenty leeches to the back.* 19*th*. Integument of the thigh rather more sensible—tongue furred—pulse quick. The skin becomes discoloured on very slight pressure, so that it is necessary to change the position of the patient frequently. The urine is ammoniacal, and deposits much sediment, on exposing which, with a drop of nitric acid, to the flame of a candle, it is evaporated to dryness, and a bright pink colour left upon the card. This test indicating the presence of lithic acid, Mr. Key ordered the patient to be kept on vegetable diet, with soda water for common drink. 30*th*. There has been little alteration, till the last two evenings, when he has been very delirious. From this time the patient became more and more emaciated—sloughs formed on the hips, and, on the 18th of August, he died.

*Dissection.* The arch and body of the twelfth dorsal vertebra were found to be fractured. The broken arch did not apparently press upon the cord, but an insulated portion of the body had been thrown backwards, so as greatly to compress the sheath. The transverse process of the first lumbar vertebra was also broken off. There was not much displacement of the column generally, and the portions of the fractured vertebra were much consolidated by the reparative process. The spinal marrow, where pressed upon, was pulpy and soft, and the remaining portion was not thicker than cream. The coverings were not affected. The bladder presented patches of ulceration, which passed through all the coats, and in the cellular membrane, at its fore-part, was a collection of pus. There was a small abscess in the cortical portion of the right kidney.—*Lancet*, 209.

*Remarks.* We know not what surgeons generally will say to the practice of *extension*, in cases of fractured spine, but we are pretty sure that Mr. Charles Bell will stare. It certainly does seem an awkward thing to put pillows under a man's belly, and then try to bend his back across them, especially if that back be already broken; but we see that, in this particular instance, if it did no good, it apparently did no harm. We apprehend, however, that all these little mechanical contrivances are not likely to be so serviceable in fractures of the spine as of the cranium. The machinery of the vertebral column is so complicated, and the parts so dovetailed one with another, that a fracture is rarely the simple thing we meet with elsewhere; and besides, the patient, for the most part, sinks, not so much from the immediate effects of the injury, as from the inflammation, and consequent disorganization, of the medulla afterwards, or from affection of the bladder.

---

### 13. Enlargement of the Upper Lip.

In a recent Number of the Journal de Progrès, Dr. Paillard has drawn the attention of his brethren to the treatment of this peculiar deformity. He observes that this said enlargement is generally considered as an effect, as well as a sign of scrofula. It usually appears in scrofulous subjects, and in that period of life when scrofula prevails—and it generally disappears when the scrofulous diathesis ceases, or is overcome. But we occasionally observe this phenomenon where there is no scrofula in the constitution, and it sometimes remains as a striking deformity, after the other symptoms of scrofula have entirely vanished.

If, in a scrofulous subject, we examine this swelling of the upper lip, we find the cellular tissue more abundant than natural, and infiltrated with a serous fluid —the muscles more pale and flabby than in a healthy subject—the skin very pallid or shining, and, as it were, infiltrated. Sometimes this swelling is covered with ulcerations, which discharge a matter that forms into scabs, which fall off, and are renewed from time to time. The cellular tissue, then, appears to be the seat of this affection. But the treatment differs, according as the complaint is an indication of scrofula, or independent of that diathesis. In the *former* case, local treatment is of no avail, and we must cure the local complaint through the medium of the constitution. In the *latter* case, the local treatment now to be mentioned is efficacious. This operation consists in an incision from one angle of the mouth to the other, along the inner side of the upper lip, and thus, in fact, dissecting away a mass of condensed cellular tissue forming the swelling. The hæmorrhage is very great; but it either ceases spontaneously, or may be restrained by ligature of the bleeding vessels. The operation is very painful, but not dangerous; and it removes a deformity which gives a peculiarly stupid cast of countenance to the individual afflicted with it. For the cases detailed in illustration of the above proceeding, we must refer to the Journal already quoted, vol. 3, 1827.

---

### 14. Baron Larrey on Fractures.

By a private communication, we learn that the above-mentioned veteran has projected, and makes use of, an extraordinary dressing for fractured limbs. He encircles the whole of the member with compresses and bandages, soaked in gummy or albuminous substances, which, on drying, form a complete, immoveable, and inflexible case for the injured limb. This he applies whether the fracture be simple or compound, and never takes it off till the cure is completed, whatever may be the degree of swelling, infiltration, or even suppuration, that supervenes. The swelling is either prevented, or subsides, without danger—the infiltrations or suppurations are absorbed—and the process of re-union goes on safely. We think it not improbable that, in simple fractures this plan may have some advantages over the splints and bandages now in use. Baron Larrey's apparatus forms a complete shell or mould, that adapts itself to every part of the limb's surface, and thus forms a more permanent and imperturbable case for the member, than any apparatus which can be constructed of splints. There is this advantage, also, that the member is never disturbed by subsequent examinations or adjustments—a frequent source of displacement of the bones. But how far this apparatus will apply to compound fractures, especially where there is much laceration of the soft parts, or shattering of the bones, we will not venture to say. We should suppose that it never can become generally applicable in such cases.

We understand, however, that some of the Parisian surgeons are adopting the plan of the Baron.—*Vide Journal de Progrès, vol.* iv.

---

15. Endermic Medication.

In a former Number of this Journal, we gave some account of M. Lembert's plan of introducing medicinal agents through the pores of the skin. M. Bally has, for three or four years past, followed up the investigation on a larger scale, and has given some short notice of the results in the Révue Medicale, for April, 1827.

He observes that, if any doubts are still entertained respecting cutaneous absorption, the endermic medication might be sufficient to dispel them. Numerous experiments have demonstrated to him the unequivocal power possessed by every part of the body's surface, for taking up, with more or less rapidity, such substances as are applied thereto. The following is a brief summary of his experiments.

The salts of morphine quickly shew their influence on the brain and nervous system, when applied to the skin. The pupils contract, and the eyes become brilliant. There is often experienced a sense of dysury or ischuria—more rarely nausea or vomiting. Sometimes a sense of pruritus is experienced over the skin. This endermic method of introducing the salts of morphine has been remarkably beneficial in neuralgic and rheumatic affections.

With belladonna applied to the insteps, our author has produced all the phenomena which that medicine is capable of, exciting when taken into the stomach, such as extreme dilatation of the pupils, and diminution of vision. The extract of squill, when externally employed, induces perspiration, increases the urinary secretion, and promotes expectoration. The strychnine has been applied through the skin, and was found to affect the locomotive system, without much disturbing the sensorial functions. In some cases of paralysis from lead, the muscular power has been restored without the production of those violent convulsive succussions, by which patients are sometimes affected under the influence of strychnine when taken internally. Our author has remarked, however, that this potent substance produces, whether taken by the stomach or through the skin, an evident turgescence about the head, and much flushing of the face, which often render it necessary either to suspend the remedy or take blood from the patient.

The deuto-chlorure of mercury, when used in this way, was attended with some unpleasant consequences on the surface, although it frequently removed severe pains in the bones and other deep-seated parts. Old syphilitic complaints were entirely cured by the endermic use of mercury. There are some difficulties attendant on this endermic medication, the greatest of which is the necessity for removing the cuticle, in order that the medicinal substance may be absorbed. In those cases, however, where there are still greater difficulties in employing remedies internally, we must not be deterred by the inconvenience of denuding the surface, for the application of the agent.

---

16. General Paralysis from Contusion on the Spine.—M. Broussais.

Capt. Dubray, aged 29 years, received a severe contusion on his back in the Russian campaign, during the conflagration of Moscow. He did not think much of the accident at the time; but shortly afterwards he began to perceive an inability to retain his urine, which inability increased. On the 15th January, 1821, he experienced, for the first time, some hesitation in his speech, and a few weeks afterwards, he felt some loss of muscular power in his hands. No medical advice was sought till October, 1822, when he consulted M. Broussais, who drew from the patient the foregoing history. M. Broussais recommended cupping, blisters, and fomentations to the spine, and a vegetable diet. This advice was not long followed, and the patient applied to another physician, who ordered a blister to his arm, and prescribed the vapour bath. This last remedy rather aggravated the complaint, and caused congestions about the head. Again, he returned to M. Broussais, and submitted to the antiphlogistic treatment and revulsives. Venesection, leeches, setons, the cautery, and vegetable diet, were prescribed. By this time the paralysis had extended far and wide. He could scarcely walk, and speech was quite annihilated. He could not retain his urine, whether sleeping or awake. The treatment made little impression on the complaint. It was merely remarked that, after sanguineous emissions, the embarrassment of speech and locomotion was somewhat diminished. The disease continuing its course, M. Dubray fell into a state of apathy the most complete, and was soon incapable of supporting the perpendicular position, or retaining the fæces. Eschars now began to form on the sacrum and hips; yet still the functions of digestion and assimilation went on well, and the patient even acquired corpulency. In the beginning of 1827, the Captain shewed symptoms of plethora, which were followed by those of gastro-enterite, of which he died in the month of April, 1827. During this last illness he shewed signs of encephalic and spinal irritation, as evinced by convulsive and spasmodic twitchings in various parts of the body, with retraction of the fingers, rigidity of the members, &c. He died on the 24th April, 1827.

*Dissection.* The arachnoid presented many traces of inflammation—the cerebral substance was injected, but not softened. The same was observable in the cerebellum and tuber annulare. The pia mater and arachnoid of the spinal marrow was injected and red, especially at the inferior portion of the canal. In the cervical and upper half of the dorsal portion the spinal marrow appeared sound, and preserved its natural consistency; but about the middle of the dorsal region, it began to soften, and this softness increased as the medulla was examined downwards, till it ended in a complete bouillie, at the termination of the dorsal region.

There was some disease of the heart and large vessels, but to no great extent. The mucous membrane of the stomach and small intestines was inflamed. The liver was turgid, and the kidneys were degenerated into a fatty mass.

---

17. Remarkable Nævus Maternus.

Mr. Bennett. Med. & Phys. Journ.

This case occurred at the Plymouth Infirmary, under Mr. Baldy, Surgeon. A male child was delivered, presenting the following appearances:—The mouth was extended to the utmost limits, and incapable of being closed, by the existence of a cluster of tumours of various sizes, which occupied and arose from the upper and middle part of the tongue. "The tumour bore, in appearance, an astonishing resemblance to a bunch of grapes, not in form alone, but also in colour." There was another nævus on the back of the thorax, resembling the wattle of a turkey-cock. The nævi were removed from the tongue six hours after birth, and little or no hæmorrhage ensued.

The most remarkable circumstances are still in reserve—the causes of these extraordinary phenomena.

"Fælix qui potuit rerum cognoscere causas."

When the woman was questioned whether or not she had longed for any particular object during pregnancy, it occurred to her that she had longed for grapes. The turkey-cock's wattle on the back was next accounted for, by the fact of her being frightened by a turkey-cock, when the pregnancy was four months advanced. As it might be thought uncourteous to doubt this explanation, we shall observe in the words of an eminent foreign writer:—"La medecine est trop éclairée aujourd'hui, pour ajouter foi à toutes les rêveries débitées sur ce sujet. On sait que ces taches, verrues, &c. sont causées par des vices d'organisation de la peau, par des alterations dans la distribution des vaisseaux sanguins sur le point altéré, (ce qui fait que beaucoup sont des tumeurs sanguines,) ou par toute autre lesion organique."

---

18. Denarcotised Laudanum.

[Dr. Hare, of Pennsylvania.]

It is now generally acknowledged, that the unpleasant effects of opium result from a principle called narcotine by the French chemists, and Robiquet informs us, that this deleterious principle may be separated from opium by digestion in æther. Dr. Hare prepares the denarcotised laudanum in the following manner.

Some opium is to be shaved, by rubbing it on the face of a jack-plane, for example, and then to be subjected, four times successively, to as much æther as will cover it, allowing each portion to be acted upon for about 24 hours. The opium left behind is then subjected to as much duly diluted alcohol as would have been adequate to convert it into laudanum of the common kind, had it not undergone the above process. The tincture thus obtained is the denarcotised laudanum. From the æther was precipitated the narcotine, or noxious principle. The digestion of the opium is conveniently done in a common Pappin's digester, and the æther should be kept near the point of ebullition. Dr. Dewees has tested the denarcotised laudanum in several unequivocal cases, when opium, in all other forms, disagreed, and he found Dr. Hare's preparation free from all bad effects.—*Philad. Journal, May,* 1827.

---

19. M. Broussais' Opinion of English Medicine.

We believe we have taken more pains to make the doctrines of M. Broussais known in this country, than any other journalists on this side of the Channel; and we must do the celebrated Professor above-mentioned the justice to say, that he returns the compliment by seizing every opportunity of making English physicians and English medicine *favourably* known on the Continent. M. Broussais is decidedly the merriest man in the merriest capital of the merriest country in the world! We have shown, on former occasions, how Tommasini and Broussais formed their judgment of medical literature and practice in this country—namely, by quoting a few eccentric or unknown contributors to monthly journals, and holding up their opinions and attainments as the standard of the whole profession! As they took their texts from quotations in the foreign journals, we supposed they were unacquainted with the English language. We are sorry we cannot, on the present occasion, offer this excuse for the *manner* in which M. Broussais erects the tribunal of criticism on this nation. He evidently understands our language—quotes direct from our journals—and signs his name to the criticisms.

The Edinburgh Journal of Medical Science (now no more) is that on which the merry Professor of the Val de Grace expends whole torrents of critical wit and satire, interlarded with expressions not quite becoming the politest nation of Europe. Dr. Brown, of Sunderland, is first put on the rack, and we should suppose he has not a single joint left undislocated! The Doctor has disputed the doctrine of fever being always dependent on local inflammation, whether of the brain or the bowels. For this he is crucified, and treated with the most profound contempt. "Que repondre (says Broussais) à un homme qui confond les affections de plusieurs organes de premier ordre dans une même denomination, sans s'en aperçevoir—qui isole les affections du systême nerveux des phénomènes vasculaires?" Again, after quoting another passage from Dr. Brown's paper, he remarks, "Est-il possible de se débattre plus péniblement dans une ontologie plus tenebreuse?"

The editors of the Edinburgh Journal, also, come in for their share of M. Broussais' censure. "Nous avons été ètonné du ton de légèreté et de mauvaise foi qui y regne. Les auteurs de ces sortes de propos ne doivent plus mêmes esperer de reussir par ces miserables moyens."

Mr. Travers next suffers. After criticising the author's doctrines of irritation, M. Broussais pounces on the editors' decision respecting the merits of the work. "A more important pathological work (say they) has not appeared since the days of John Hunter. Mr. Travers is the *first* who has successfully drawn the line of distinction between the phenomena of irritation and those of inflammation, which have been so long confounded together." Upon this passage the following terrible judgment is passed—and the whole of the English faculty is, at one fell swoop, consigned to damnation! "Evidemment le *mal est radical* chez ces Anglais: tant qu'ils se contenteront d'expressions vagues et générales, tant qu'ils ne sentiront pas la necessité d'aller chercher l'organe malade—c'est-à-dire tant qu'ils seront ontologistes, *ils ne comprendront rien à l'importance de la medecine physiologique.*"

Dr. Gairdner is then shewn up. Our readers know that this gentleman has published some cases of erosion or perforation of the stomach, on which we were induced to pass some strictures, at page 87 of the sixth volume of this series. The inconsequences of Dr. Gairdner were not likely to escape the eagle eye of the French critic. "Behold," says he, "the kind of reasoning which Dr. G. uses. There existed, during life, the symptoms of an affection of the stomach—after death there was found a lesion of structure in the organ—*therefore*, the lesion did not take place during life, but after death." We must confess, we are unable to parry the satirical shafts of the Parisian Doctor on this point.

The practice of curing gonorrhœa by camphor, as recommended by Mr. Bell, of Edinburgh, "gives," says M. Broussais, "an excellect idea of the state of medicine in England." This is not fair. In England, as in other countries, there may always be picked out eccentric or ridiculous notions and practices, the onus of which should lie on the shoulders of the individuals, and not be fathered on the whole country. M. Broussais details the case of a physician, communicated by the patient himself to the editors of the Ed. Journal, and which was treated with camphor and aperient medicines; concluding with this remark:—"Ainsi voila une urethrite qu'*une application ou deux* de sangsues, et l'usage des autres antiphlogistiques, auroient enlevée *en trois jours*, qui a fait souffrir le malade pendant près d'un mois." If M. Broussais is always able to cure a gonorrhœa in *three days*, by the application of a few leeches and antiphlogistic regimen, we must say he is very clever. He certainly would make a fortune in London by such practice!

Here we shall stop. It will probably be our own turn next to come under the lash of the renowned founder of the "Doctrine Physiologique." We shall neither deprecate nor provoke his ire; but continue, as we have done, to hold up what we consider to be good in his doctrine for the information of our countrymen, not fearing, at the same time, to object to what appears to be fanciful or erroneous. We ask no more in return from M. Broussais. But we would just suggest to that illustrious Professor that, before he judges of a whole nation or profession, he should be well acquainted with the literature of that nation or profession—and not visit the sins of a few on the many. It is possible that the Medico-chirurgical Review has hitherto escaped the censure of the acute Frenchman, because it does not permit these eccentricities to pass without comment in its pages—an advantage which it could not possess if it published original communications in the usual manner. This advantage we have not yet seen any good reason to forego.

---

### 20. Blindness from a Blow on the Fifth Pair.

*Case.* W. Carter, aged 38, was received into Guy's Hospital, on the 10th April, in consequence of a severe blow on the right superciliary ridge of the os frontis, by the handle of a whip, followed by tumefaction and ecchymosis. On examination it was ascertained that the iris was not lacerated, nor was there any turgidity of the humours. There was much pain about the supra-orbital foramen, extending in the line of the nerve on the forehead. The power of vision was entirely lost in that eye—the pupil dilated—the iris entirely insensible, as was the retina. By cupping, purging, and mercury, carried to ptyalism, the transparency of the humours (which had become a little turbid) was restored, but vision appears to be lost for ever.—Dissector.

If the blindness, in this case, results entirely from the blow on a branch of the fifth pair, and without any direct injury to the humours, retina, or optic nerve, it is very curious. But, for our own parts, we do not think this is the case. We conceive that the retina was injured by the blow.

---

### 21. New Method of treating Syphilis. By Dr. C. H. Dzondi, Professor in the University of Halle.

Professor Dzondi, from an experience of ten years, comes to the conclusion that the best method of treating syphilis, is not by small doses of mercury gradually introduced into the system, but by large doses quickly administered. The fundamental principles of this new method are these:—1mo. That mercury is a dangerous poison, the effects of which are much more difficult of cure than the most inveterate syphilis, 2ndo That mercury is indispensable in the cure of the disease, in all cold countries; and is only poisonous when exhibited in a certain manner. 3tio. That the present mode of administering the remedy is, in general, inadequate to the complete eradication of the disease, except in cases where the virus is mild. In cases where it is otherwise, it either aggravates the malady, palliates the outward symptoms, or masks the disease from view. 4to. That the oxymuriate of mercury, properly prepared, is the best form of the remedy. Professor D. begins with about half a grain of the sublimate for a dose, (per diem,) and increases it to two or three grains daily. He is convinced that a large quantity may be taken in small doses, without ultimate cure; while a small quantity given in large doses will be speedily effectual. He gives the medicine immediately after taking food, and never on an empty stomach; sometimes combining opium with it, when pain is complained of in the stomach or bowels. He does not consider it necessary to make any change in the *kind* of food which the patient takes during the course. Salivation rarely takes place under this mode of treatment. The duration of the course is generally four weeks, during which, the action of the skin is to be promoted by warm air, warm cloathing, and confinement to the bed-room in cold weather. In Summer, and in very fine weather, the patient may be permitted to go out for an hour or two in the middle of the day. In order to quicken the action of the absorbents, and thus to diffuse the remedy as rapidly and as extensively through the system as possible, the patient is to take no more food than is absolutely necessary for the support of life. He does not particularly object to alcoholic, or other stimulating drink, in moderation. Sarsaparilla he considers the best auxiliary to the mercury; but by no means adequate to the cure of the disease by itself, especially in northern climes.

The above practice appears to be a modification of that which has been tried and recommended by some surgeons in this country—we mean Mr. John Cunningham and Mr. James Boyle, surgeons in the Royal Navy. They gave calomel in 20 grain doses twice or trice a day, so as rapidly to affect the system, when the syphilitic symptoms were found quickly to give way. We leave it to our surgical brethren to think on these proposals, based as they are on experience. The high character of Professor Dzondi guarantees the authenticity and veracity of any thing proceeding from his pen.

---

### 22. Purpura Hæmorrhagica.

A case of this kind is published by Mr. Kingsley, of Roscrea, in the Lancet, No. 199. The patient, a man 38 years of age, stated that he had a constant discharge of dark-coloured blood from the mouth and throat, which appeared to exude from the mucous membrane, the gums being covered with sordes, and coagula of blood being seen in various places. The upper and lower extremities were sprinkled with spots, of various sizes and hues, from a very dark purple to a log-wood colour. There were but few of these on the body. Some of the petechiæ had scabbed, and formed ulcers with a yellow surface. The pulse was 80, small and soft—skin cool—bowels and urine free, debility great—good appetite—no thirst nor fever. He had had three attacks previously, and said he was cured by bark and elixir of vitriol. The same plan was now adopted; but the result was an increase of the disease, with appearance of blood in the stools and urine. The pulse was 100, firm and hard. He would not consent to be bled, and Mr. K. could only discontinue the tonics, and prescribe low diet, oranges, &c. with five grains of blue-pill at night, and a mixture of oil of turpentine and castor oil every morning. Digitalis was given through the day. No improvement took place under this plan, and it was discontinued, with the exception of the turpentine. The bark and acid again given. He still continued to get worse, and at last consented to be bled, and was put on the most antiphlogistic diet, with vinum colchici every four hours. The next day he was better. The blood was much inflamed. Bled again to twelve ounces, and the bowels to be kept open with castor oil. In two days more, the discharge of blood had ceased from all the outlets. The mouth was sore from the mercury, and considerable ptyalism ensued. While this lasted, there was no return of the hæmorrhage; but there was a relapse, which continued several days, and ultimately disappeared, under treatment similar to that which has been stated.

Mr. Kingsley has made some sensible remarks on this mysterious disease, which often baffles every mode of treatment, and then goes off spontaneously. We have succeeded, and we have failed, with the most opposite plans of treatment. We believe, with the able and philosophic Dr. Dawson, (Nosological Practice of Physic, p. 246) that the disease is sometimes sthenic, sometimes asthenic—and, consequently, requiring different remedies in different cases.

---

### 23. Bronchotomy.

Dr. Heustis, of Albania, was called to a child, three years old, who was said to be dying, in consequence of a grain of corn having stuck in its throat about a week previously. When Dr. H. arrived, he found the child suffering under impeded respiration, cough, and other symptoms of an extraneous body in the trachea. Dr. H. proceeded to the operation, and was a good deal embarrassed by the discharge of venous blood. Having exposed the wind-pipe, he carefully made a longitudinal incision, half an inch in length. He now sought for the foreign body, but without success. He pushed up a director, till the point of it surmounted the rima glottidis. Nothing could be found, and, therefore, the wound was simply dressed. The little patient seemed relieved. Two days afterwards, the grain of corn presented itself at the artificial opening, and was removed, without having undergone any material change. The relief, in this case, before the foreign body came to the wound, must have resulted from the partial breathing through the artificial opening.—*N. York Med. and Phys. Journal, Jan.* 1827.

---

### 24. Physiological and Pathological Results of Extirpation of the Kidneys.

By Professor Mayer, of Bonn.

Passing over the crude speculations of the ancients, respecting the functions of the kidneys, and the rôle which this function occupies in the animal economy—passing over, also, the instances in which these organs were found wanting in fœtuses and in monsters, we shall come at once to modern times, when experiments have been made with care, and their effects accurately noted.

When Richerand extirpated one kidney of an animal, no inconvenience appeared to ensue; but, when both kidneys were removed, a morbid condition obtained, and death took place in a very few days. In all Richerand's experiments, the gall-bladder was found gorged after death. The principal phenomena which succeeded the ablation of both kidneys, were:—Vomiting, tremors, smallness of pulse, urinous odour in the liquids vomited, borborigmi, intermissions of the pulse, coldness of the body—death in three days. On dissection, some effusion was found in the abdomen, but no inflammation—venous system gorged with blood—no alteration in the chest—slight effusion into the cerebral ventricles. In some cases, the animal died in a quarter of an hour after the extirpation, and nearly the same symptoms and *post-mortem* appearances presented themselves.

Prevost and Dumas made similar experiments, and they discovered the presence of urea in the blood, after renal ablation. M. Mayer made a number of experiments, of which he has detailed ten, in a late Number of a French journal. We shall only give the results, and not the details.

1. The extirpation of both kidneys causes inevitable death of the animal, at various intervals.

2. The principal phenomena observed, were, tremors; crying, apparently from internal pains; and, finally, convulsions and death.

3. There were no well-marked symptoms of abdominal inflammation.

4. The operation is followed by the secretion, in various organs of the body, of a fluid, having all the physical characters of urine. This secretion takes place, particularly in the abdomen, chest, pericardium, ventricles of the brain, the eye, stomach, and intestinal canal. It even takes place in the cellular tissue of the liver, lungs, muscles, testicles, &c.

This urinous serum was submitted to chemical analysis, and our author's experience coincided with that of Prevost and Dumas, who also detected urea in the blood of animals, after ablation of the kidneys, corresponding with the fact, that men, whose kidneys have been affected with organic disease, have vomited up matters clearly of a urinous character. We cannot, then, says our author, deny, that a urinous liquid may be formed, under the above circumstances, in various other parts, besides the kidneys—but particularly in secreting structures.

Dr. Mayer thinks it probable that the cause of death, after these experiments, is owing to the irritation of the brain, from the urinous fluid thrown out there. —*Journal Complémentaire.*

---

### 25. On Digestion.*

The offer of a prize by the Parisian Academy, a few years ago, for the best experimental essay on the subject of digestion, drew forth two competitions:—one by Tiedemann and Gmelin, in partnership—the other by Leuret and Lassaigne, in similar co-operation. Neither of the Essays obtained the prize; but both were thought so meritorious, as to be rewarded, each, with a donation of 1500 francs.

Although the industrious experimenters have been unable to clear up the almost mysterious process of digestion, yet they have thrown much light on many of the agents and agencies employed therein by mother Nature. In an article of this kind, we cannot be expected to go into the analytical operations by which the authors arrived at their conclusions—we can only exhibit an abstract of the *results* of their experiments and researches.†

1. *Mastication* being the first step of digestion, the properties of the *saliva* naturally engaged the attention of the candidates. This fluid was found to be nearly identical in all animals—to contain about one per cent of solid matters, consisting of soda, muriate of soda, muriate of potassa, carbonate of lime, phosphate of the same, a very trifling quantity of albumen, and a great deal of mucus. In the sheep, there was found a sensible quantity of *sulpho-cyanic acid*, one of the most deadly poisons—and the German physiologists proved its existence in the saliva of man. Both the German and French authors have repeated former experiments on saliva, and they agree that it accelerates solution, or rather putrefaction; but they seem to consider its chief use to be that of lubricating the mouthfuls of food, rendering sapid bodies amenable to the gustatory nerves, and preparing for the process of digestion, by *softening* the aliment. The German physiologists hazard some other conjectures as to the uses of saliva, as that its animal constituents promote the assimilation of *unazotized* matters of food, &c. but these are mere speculations.

2. *Chymifaction.* This is taken in a wider sense than we are accustomed to take it. Our physiologists consider chymifaction as the whole of the process which prepares the food for entering the chyliferous vessels—consequently including the actions of the stomach and its juices, the liver, the pancreas, and the glands of the small intestines. The candidates necessarily examine into the separate parts which the above organs play in the mysterious drama of digestion.

3. *The Gastric Juice.* The German physiologists have taken immense pains in the investigation of this interesting subject. They inform us, that the succus gastricus of a fasting stomach, is a clear, ropy, and opake fluid, nearly, if not quite, destitute of acidity. But, if the organ be stimulated by any, the most simple agent, then the secreted fluid is constantly acid, and that in proportion to the stimulation employed. The French experimenters, on the other hand, maintain that the gastric juice is *always* acid, and that its component parts are, muriate of ammonia, chloride of sodium, mucus, a peculiar animal principle soluble in water, phos-

---

* Leuret, Lassaigne, Tiedemann, Gmelin.

† In the construction of this article, we have availed ourselves, in addition to the original works, or their translations, of some extensive analytical portraits of the Essays in question—one in the Journal Complementaire, for September, 1826—another in the Revue Medicale, for May and June, 1827—a third in the Edinburgh Journal, for April and October, 1827, and a fourth in the Nouvelle Bibliotheque Medicale, Mai, 1827.

phate of lime, and *lactic* acid. They deny the accuracy of Dr. Prout, in his inference, that free hydrochloric acid is disengaged during digestion. The Germans make the gastric juice, as obtained from the horse and the dog, to consist of mucus, osmazome, salivary matter, alkaline sulphates and muriates, (the alkali generally soda) phosphate and muriate of lime, with some other unimportant ingredients. They maintain, contrary to Leuret and Lassaigne, that the acidity (in the dog) is owing to muriatic and acetic acids—to which (in the horse) is added the *butyric*. They support the deductions of our countryman, *Prout*.

Both parties agree that, when the gastric juice is secreted in consequence of the stimulus of food, the chymous mass is always acid, the Germans maintaining that the acidity is greater, in proportion to the indigestibility of the food. Thus, in dogs and cats, the acidity was greatest when they were fed on albumen, fibrin, bones, gristle, and the like, while it was less when the aliment consisted of starch, gelatin, potatoes, rice. When fed with liquid albumen, the gastric juice contained nearly enough of alkaline ingredients to neutralize the acid. The quantity of gastric juice secreted during digestion was found to be much greater than people are aware.

As to the power of the gastric juice, in dissolving substances out of the body, both German and French agree, with Spallanzani, Gosse, and others, that it has such power, contrary to the deductions of Montegre, and the French school generally. The solvent properties of the succus gastricus would now seem to be indisputable. The German physiologists endeavoured to ascertain whether this solvent power of the gastric juice could be explained by the action of the different constituent parts, separately, on substances. Their experiments are not complete, and they do not appear to have tried to imitate the gastric juice by a *combination* of all its component parts.

4. *The Muscular Power, or Churning Process of the Stomach.* This is attributed by the German physiologists to the influence of the par vagum—and they appear to attach no other influence or power to these nerves, in the process of digestion, than that of moving forward the digested layers of food towards the pyloric orifice of the stomach, thus permitting new portions of the alimentary mass to come in contact with the coats of the organ. The younger Legallois, who reviews the works above-mentioned in the Revue Medicale, comes to the same conclusion, in opposition to the experiments of Dr. Philip. We see, from this, how difficult it is to come to positive conclusions where muscular power and gastric juice are both necessary, in this way, to the digestive process. Before quitting this part of the subject, we may allude to the curious phenomenon observed by M. Gendrin, when the eighth pair of nerves were divided—namely, inflammation of the mucous membrane of the stomach. Will this throw any light on the inflammation which takes place in the mucous membrane of the bladder, when the spinal marrow is compressed, or otherwise materially injured, as we generally find to be the case ?

5. *Intestinal Digestion.* In proceeding to this part of the subject, it is necessary to revert to the secretions poured forth by the organs auxiliary to digestion, as the pancreas and liver. The succus pancreaticus has been carefully attended to by both the German and French experimenters. They all agree that the *quantity* of this fluid is very small. The French say it is always alkaline ; while the Germans aver, that what is collected *at first* is freely acid, and afterwards becomes faintly alkaline, the change being ascribed to the perturbation occasioned by the operation. In respect to composition, Leuret and Lassaigne consider it similar to the saliva, and this, indeed, is the general opinion of physiologists. But Tiedemann and Gmelin differ materially from the French physiologists on this point. They affirm that the succus pancreaticus differs from saliva, in never containing sulpho-cyanic acid, free soda, or mucus—in being acid in its natural condition—and in containing a larger proportion of albumen. The fact is, as Magendie has stated, that nothing is known of the purpose which the pancreatic juice serves in the process of digestion.

6. *The Spleen.* Messrs. Leuret and Lassaigne have made some interesting experiments in regard to the function of the spleen, seeming to lean to that theory, as the most probable, which considers this organ as a diverticulum for the blood during digestion. We know that when the stomach and intestines are distended with food, there is an increased afflux of blood to the whole villous membrane of the alimentary canal, and, therefore, an additional quantity of blood to be returned through the portal vessels. But, as these are not well calculated for forwarding this mass of fluids, it follows that the meseraic veins must become gorged—unless the splenic vein ceases to discharge the usual quantity into the vena portæ. A diminution of its discharge is probably effected by a distention of its minute ramifications. The French experimenters found that a dog's spleen, which usually weighs but a few ounces, weighed 24 ounces, in two hours after the application of a ligature to the vena portæ. In most animals, the spleen has a rosy tint, when they are fasting—becoming of a blueish colour when chymification has begun, and acquiring a blueish *black* colour and turgidity when the chyme has passed the pylorus. The theory is specious, and not entirely new; but it is far from being unexceptionable.

7. *The Liver.* The LIVER and its secretion are next to be considered, as auxiliaries in the work of DIGESTION—and, as many of our modern doctors would say—in the work of INDIGESTION.

The BILE has been analyzed by the French candidates, and its composition was found to agree very nearly with the analysis of Thenard, (resin, picromel, and yellow colouring matter, as organic ingredients, and phosphate, muriate, and sulphate of soda—muriate of potass, phosphates of lime and magnesia, free soda, and a little iron, as saline ingredients,) together with cholesterine discovered after Thenard's analysis. The German physiologists, however, have discovered several other organic principles in the bile, shewing it to be one of the most complex of the animal fluids. They nearly agree with Thenard and the French candidates, as to its saline ingredients; but they consider the picromel of the former as a compound of resin of bile and a crystalline principle, possessing all the properties of sugar, except that of fermenting—and containing azote, so as to resemble exactly the sugar of gelatin, as procured by Braconnot. They still allow this compound, which has the property of rendering the resin soluble in water, to retain the name of picromel. In addition, they have discovered the existence of *asparagin* in the bile, and this substance appeared in the form of colourless crystals, soluble in sixteen parts of water—not soluble in alcohol—very soluble in nitric acid. This biliary asparagin has also the property of rendering the resin of bile soluble in water. Besides the above principle, they have also discovered in bile, the mucus of the gall-bladder, casein, albumen, gliadine, (that part of glutin soluble in alcohol,) a little osmazome, oleic acid, margaric acid, acetic acid, and a new, or *cholic* acid. They have made many other discoveries respecting the component ingredients of the bile, which we think it needless to record. Enough has been said, to shew that the bile is a compound which the chymist will not easily imitate! We shall state one observation on the means of detecting bile. When nitric acid is added in small successive portions to fluids containing bile, it causes first a green, then blue, next violet, and, lastly, a red colouration, which becomes yellow on standing, or on the addition of a large excess of acid.

*Use of the Bile.* Our readers are well aware that Mr. Brodie made some experiments, a few years ago, from which he was led to infer that the bile was necessary for *chylification*, since no chyle could be found in the intestines or lacteals when the excretory duct of the liver was tied, although the ligature did not prevent *chymification* in the stomach. The results of Mr Brodie's experiments were confirmed by Mr. Mayo. Unfortunately for experimenters, the researches and operations of both the German and French physiologists have given the negative to the experiments of Brodie and Mayo, as they have found the chylifactive process go on in those animals whose biliary ducts were secured by ligature. The German candidates remarked that the animals so operated on, were first attacked with vomiting soon after the ligature was applied—then with thirst and aversion to food. On the second or third day, the eyes became yellow—stools chalky and fetid—urine yellow. Some of the animals died—some were killed. Of the latter, some had recovered from the jaundice—owing to a remarkable phenomenon observed also by Mr. Brodie, the re-establishment of the obstructed bile-duct. They observed that chymification went on as before; and, in the small intestines, they found nearly the same principles as in sound animals, with the exception of those derived from the bile.* The contents of the great intestines were also nearly the same as in healthy animals, excepting the absence of the biliary principles; but these fæcal contents emitted an exceedingly fetid and unpleasant odour. The lacteals and thoracic duct contained abundance of yellowish fluid, which coagulated like ordinary chyle, the crassamentum acquiring the usual red colour—in short, the only difference was, that in the animals experimented on, the chyle was never found white. The reason which they assign for this is, that where the bile is obstructed, the fatty matters of the *chyme* are not dissolved and mixed with the rest of the fluid, thus giving it the white colour in healthy animals. This explanation seems supported by the fact that, when animals are fed on food containing no fat, the colour of the chyle is not white. The German professors, then, confine the use of the bile (as far as chymification is concerned) to the solution of fatty matter in the chyle. We do not think that they are by any means authorized to draw such a conclusion. They go on, however, to other supposed uses of the bile. They attribute to it a stimulant property, by virtue of which it excites the flow of the succus intestinalis, as is pretty clearly proved by the dry state of the intestines in jaundiced subjects, and in animals whose bile-ducts have been tied. They also think it probable that the bile stimulates the intestinal muscles into action. Thirdly, it may contribute to azotize or animalize those articles of food which do not contain azote. Fourthly, they believe that it tends to prevent the putrefaction of food during its course through the intestines. Fifthly, it tends to liquify and dissolve the fatty matters in the food. Lastly, the physiologists in question are disposed to allow that the bile is an important *excretion*—and that the liver is highly useful in throwing off a considerable proportion of carbon, which is not thrown off by the lungs in a state of oxidation. The authors endeavour to support this doctrine by many ingenious arguments drawn from anatomy, human and comparative, as well as from physiology and even pathology. But we need not pursue this subject any farther. Those who attentively observe the phenomena presented to their view, both in health and disease, are well aware of the important part which the condition of the bile plays in the animal economy, in both the above states.

8. *Succus Intestinalis.* The German professors observed that, in animals which had fasted long, there was seen, on the inner surface of the intestines, a thin layer of firm mucus, of a faint yellow colour. They also observed that, if pebbles or pepper had been swallowed a little before death, a quantity of thinner ropy mucus, and an augmented secretion of bile had been poured out. The French physiologists, moreover, observed that, when the villous coat of the duodenum was exposed and cleaned, and then touched with diluted vinegar, the membrane immediately exhaled a clear fluid, while the biliary and pancreatic ducts discharged much bile and pancreatic liquor. It is evident, however, that the exact composition of the succus intestinalis can never be correctly ascertained, as it cannot be obtained free from admixture of other

* Legallois, in reviewing this part of the German work, observes that Magendie found the chyle white in two instances after ligature of the biliary duct; but that the attendant circumstances are not sufficiently detailed. The French physiologists took especial care that the animals had fasted a long time after the ligature, and that their bowels should be well cleared before the new food was introduced. Under these precautionary measures, the thoracic duct was found distended with a yellowish fluid nearly transparent, and having a saltish taste. The experimenters regarded this as genuine chyle; but Legallois remarks: "Cependant il faut avouer que son analyse chimique le rapproche singulièrement de celle de la lymphe, telle que l'a donnée M. Chevreul: il n'en differe essentiellement que par une difference de quelques centièmes dans la quantité de la fibrine."—Rev. Med. p. 258.

secretions. Both classes of experimenters believe that it possesses the power of dissolving the food. There cannot be a doubt that a finish is given to the assimilation of chyme as it passes along the intestines. It was ascertained that the acidity of the chyme diminished as it descended lower in the bowels, and that it entirely disappears in the cæcum.

9. *The Chyle.* In respect to the CHYLE itself, there is not much to be said. The German physiologists deny that it exists at all in the intestines; while the French maintain that it may be found even in the stomach. They all agree as to the material chemical ingredients in that fluid. Dr. Marcet's analysis is substantially corroborated. The chyle is known to consist of two portions—serous and fibrinous; the latter separating, like that of the blood, by spontaneous coagulation. The firmness of the coagulum would seem to depend, in a great measure, on the quantity of fibrine. Chyle, however, scarcely coagulates before it has passed the mesenteric glands. After that, the fibrine begins to appear, and is much more abundant after the addition of the lymph from the spleen, which contains a large proportion of fibrin. The quantity is lessened in the chyle of digestion—and increased in the chyle formed after ligature of the ductus communis choledochus. It abounds in the lymph from the lower extremities.

In like manner, the chyle contains no red particles before passing the mesenteric glands; but does so immediately afterwards—and more especially after mixing with the lymph from the spleen. The chyle frequently contains fatty matter—but little or none after fasting, or if the animal is fed on matters not containing fat—most of all, when the food is very fat; when, for example, butter is mixed with it. This fatty matter does not appear to be dissolved, but exists in a state of minute division and suspension, giving the chyle its peculiar white colour. The serum of the chyle is generally alkaline. From the above and other facts, the German physiologists infer that the fibrin, the colouring particles, and the albumen of the chyle, are supplied either not at all by the intestinal lacteals, or at least, in much less quantity than by the lymph, which is formed by the blood; that the food supplies chiefly fatty matter and other principles soluble in alcohol, especially osmazome. These inferences are liable to some objections; but we have not time or space for going into the subject farther in this place.

In conclusion, it will be remarked that, although no brilliant discovery has been made, yet many interesting facts have been brought to light, which may ultimately bear on the practice of medicine as well as enlighten our views of the animal economy. On this account, we have endeavoured to draw up an exposé of the labours of the above-mentioned distinguished physiologists in as concise a form as we possibly could; and hope that no very material facts have been omitted, short as our analysis has necessarily been.

---

### 26. Pythagoras Redivivus.

Sir George Gibbes has published a short paper in the November number of the Medical and Physical Journal, the tendency of which is to revive the ancient doctrine of Pythagoras. It has long, indeed, been admitted, that no particle of matter can be annihilated. It may change its form, but still it is matter in some shape or other. Sir George considers it well ascertained, "that all the animal tissues are resolvable on decomposition into minute bodies, which, in water, and under the influence of the sun, possess life and activity." These animalcula, or ultimate points of vital activity, cannot be further decomposed, except by fire, which renders them amenable to chemical laws, and changes them into gas.

The vitality and activity of the INFUSORIA depend on the influence of the sun, under which every pool of water becomes tenanted by myriads of them. The sun, then, the source of life as well as light, supplies the vitality in all the endless variety of organised and living action, "and modifies matter, in all these processes, in a manner totally different from all physical and chemical principles." In the dissecting room, our author observes, vitality is not lost, although the life of the individual is gone; "for every part of the organized structure resolves itself into new arrangements, and myriads of vital rudiments re-assert their rank in the living world."

This is precisely the doctrine of Pythagoras, as versified by Darwin.

"——————the restless atoms pass
From life to life, a transmigrating mass."

"Thus, manures supply them (living animalcula) to the growing vegetables, and digestion prepares them for the use of animals." Thus, the human fabric is built up by innumerable myriads of living rudiments, with powers totally different from those of chemistry or physics.

More than 30 years ago, our author instituted a series of experiments, that appeared, then and now, conclusive, as to the essential purpose which the animalcula infusoria perform in the growth of vegetables. He examined with a microscope the green matter which forms on water, the animalcula infusoria, and the fibrillæ of the roots of other vegetables, whilst growing in water. "Myriads of animalcula," says he, "may be seen around the extremities of such vegetables, and it appears that these minute living bodies agglutinate themselves together, and *absolutely themselves become the added part;* so that the fibres seem to be nothing more than a congeries of these animalcula, forming the growing part." Again:—"If a bason of water be half shaded from the sun, whilst the other half is exposed to its rays, we find the shaded water to be without animalcula, whilst they swarm by myriads in the exposed portion." If a sprig of mint be placed in this water, the fibres of the roots extend and grow in the illuminated portion, but make no advance in the dark part. The animalcula are seen to be supplied on the one side, and to fix themselves on the ends of the fibres, increasing them longitudinally. On the other side, the animalcula being absent, the roots do not grow. The increase of the several parts of vegetables seems entirely dependent on the supply which they receive of these animalcula by the roots, leaves, &c. "for the leaves and blades of corn, even when growing in a room, are terminated by drops of water, evidently supplying these monades, which arrange themselves according to the necessities of the growing vegetable, and according to the impulse originally given, and continually supplied by the seed of the plant."

These views are certainly very ingenious, and by no means improbable. We shall be glad to see Dr. Gibbes's application of them "to the actions and functions of the animal system."

---

### 27. The Instrument of Justice.

In the November Number of the Medical and Physical Journal, Mr. Mayo, surgeon to the Winchester Hospital, has related an interesting case of a fleshy tumour, occupying the greater part of the temporal fossa of the right side, in a young man, on whom Mr. Bell had previously operated at the Middlesex, without success. The tumour had an elastic feel, and was now disposed to protrude the ear. The patient was anxious for an operation, and Mr. Mayo attempted to remove the swelling by excision; but, finding it adherent to the cranium, which felt rough, as if from absorption of the external table, he cut away as much of the diseased mass as he could see or feel, and then filled the cavity with lint, dipped in oil of turpentine. In about a week, a fungous substance, resembling the original tumour, began to protrude, and various escharotics were employed, to check the morbid growth, without effect. Under these circumstances, a consultation was held, and it was deemed advisable, as a dernier resort, to tie the carotid artery, and, by thus cutting off a great supply of blood, to arrest the progress of the tumour. The operation was performed without accident; but the advantage was only trifling and temporary. The tumour slowly increased, and sometimes bled profusely. He became affected with fits, resembling epilepsy, and was finally worn out.

On dissection, the brain was found adherent to the dura mater, at the part, and, when peeled off, (from within) the pia mater and substance of the brain appeared to have been absorbed by the pressure of the tumour, which now presented itself, passing through a portion of the temporal and parietal bones, and occupying the temporal fossa of the sphenoid bone within the cranium, still closely covered by the dura mater. The tumour weighed about two pounds, appeared to be of uniform consistence, and resembled pancreatic sarcoma. The morbid growth is preserved in the Windmill-street Museum.

And now for the INSTRUMENT of JUSTICE—the LIBERAL JOURNAL! The case is thus *fairly and properly* introduced. "Editor Roderick, with considerable generalship, has placed the account of this immediately previous to Mr. Earle's case, *in order that the reader might be led on, step by step, from one USELESS operation to another.*" The *whole account* of the case is then given, in the *minutes of dissection*, without stating one single iota of any thing that happened previously to the *post-mortem* examination—thus *judging* a medical practitioner, not by the events which occurred, or the phenomena which presented themselves *during the life* of a patient, but *solely* by the appearances *on dissection!!* We appeal to every honest man in the profession, whether the annals of the Inquisition, or the tribunals of Turkish despotism, ever presented so diabolical a judicial proceeding as the above! Yet, this is the Journal which boasts of its *independence*, while it proves itself, in every number, the fiend-like tool of a faction, without a single spark of judgment, justice, or liberality, in its composition. But, henceforth we shall be in a position to *quickly* disseminate the antidote with the poison of these scandalous delinquencies, and flagrant violations of equity and truth—by which we hope "to do the state some service."

---

28. SECTION OF THE PNEUMO-GASTRIC NERVES.

[M. DUPUY. Veterinary School of Alfort.]

It is now ascertained, beyond all doubt, that section of the par vagum, on both sides, destroys an animal, say the horse, in a few hours, by paralyzing the muscles about the larynx, and thus causing asphyxia. When death is thus produced, we cannot properly ascertain the real effects of the interruption of the nervous influence on the stomach, lungs, and other organs. M. Dupuy, therefore, in his experiments, opens the trachea of the horse, by which the animal is made to live from fifty to sixty hours. The gentleman in question has laid some experiments lately before the Medical Society of Paris, in which the effects of the pneumo-gastric section are shewn. Thus, the nerves were divided in both sides of a horse's neck, and portions cut away. Tracheotomy was then immediately performed. The animal was carefully examined at certain periods, and also bled before and after the operation, in order to ascertain the effects of the section on the blood, as well as on various functions. Two hours after the section, there was no alteration in the functions of respiration, circulation, &c. The animal continued to eat as before. In four hours, the breathing was accelerated—the blood from the carotid was now darker in colour than before—the animal ate and drank, but deglutition seemed performed by a convulsive action, and liquids returned by the wound in the trachea. At the end of 16 hours, the breathing was slow and deep—much mucus flowed from the wound in the trachea, and the food returned by the same aperture—the action of the heart is much weakened—arterial blood is now nearly as dark as venous. The fourth examination, at the end of 28 hours, shewed no material alteration. At 40 hours from the operation, the animal had great difficulty in swallowing—the blood from an artery was quite black—the œsophagus was felt to be crammed with food—and the horse was comatose. At 52 hours, the breathing was stertorous, and he soon died.

*Dissection.* There was nothing wrong in the brain. All the parts forming the aperture in the larynx were swelled and red, so that the passage was almost entirely closed. The mucous membrane of the larynx and trachea was red, and otherwise discoloured, but these were considered cadaveric phenomena. The lungs were inflamed and hepatised—the bronchia were filled with mucosities. The substance of the heart was much softened, and its cavities filled with black blood. The œsophagus was filled with alimentary matters, as were the pharynx and nasal cavities. The stomach was filled and distended with food almost dry, and adherent to the mucous membrane of the organ, which was of a red colour. There was no chyme in any of the intestines. The liver was enlarged—and there were several large black spots on the spleen.

From the above, and other experiments of a similar kind, it is evident that section of the pneumo-gastric nerves stops the sanguifactive (arterializing) process in the lungs—paralyzes the œsophagus—and puts an end to the secretion of gastric juice, and, consequently, digestion, in the stomach.

The experimenter found, in this, and several other cases, that a peculiar effect is produced on the spleen by these operations—namely, that its blood is changed, and capable of *producing* a disease in other animals, when introduced by a puncture, which affects *their* spleens and causes death. The same was found to be the case with the spleens of animals living in marshy countries, where intermittents prevail. This is a very curious and interesting fact, which seems to be corroborated by one related by M. Adouard, and which we shall append to this paper.

### Pemphigoid Affection of the Spleen, producing a Contagious Material.

By M. Adouard.

The following case was published at the time of its occurrence in the Annals of the Medical Society of Montpellier. It is here republished, for the reasons above-mentioned.

A French cavalry soldier was affected for some time with ague, in the malarious countries about Lodi. When M. Adouard took charge of the Military Hospital, this man was cured of the ague, but laboured under a considerable enlargement of the spleen. This was much reduced by proper means, and the patient was nearly re-established in general health, when he suddenly expired one morning, without any thing to account for the fatal event.

On dissection, nothing could be discovered except the enlargement of the spleen, on the surface of which, were several phlyctenæ, of different sizes, elevated, nearly colourless, and containing a yellowish fluid, like that of pemphigus. A drop of this fluid came in contact with a chap on one of M. Adouard's fingers, and caused instant stinging pain. This was followed, in eight hours, by such constitutional disturbance as to force him to quit a company in which he was spending the evening, and retire to bed. At midnight, he had a severe rigor of two hours' duration, followed by intense fever, and, ultimately, by perspiration and comparative apyrexia. Next day, there was an ugly looking pustule on the part affected, and his whole mental and physical powers were prostrated. Great swelling and inflammation of the hand and arm followed, with fever of a *remittent character*, but extremely severe. On the 7th day, he was delirious, and a copious perspiration solved the fever. A large suppuration had formed among the muscles of the fore-arm, which was opened, and then the patient rapidly convalesced.

*Journal Général.*

The above case may be considered, by many, as analogous to those of dissection wounds; but, if M. Dupuy proves that blood or the contents of phlyctenæ in the spleens of animals produce, by inoculation, similar diseased states of spleen in other animals, as he pledges himself to do in a Memoir about to be published, the case will assume an importance beyond that of common dissection wounds. We shall lay before our readers an early account of the promised Memoir.

---

### 29. Fatal Exhalation of Blood into the Stomach.—(M. Adouard.)

Pathological anatomy has clearly proved that great effusions of blood may take place into the substance of the brain and the lungs—or into the cavities of the viscera, as well as of the thorax and abdomen, by a kind of exhalation, leaving no trace of ruptured vessel. The following is a remarkable example.

A soldier, of lofty stature, and sanguineous temperament, in the prime of life, became affected with tertian fever at Rome, during the French occupation, and was in the Military Hospital for cure. One day, when it was the period for the return of a paroxysm, he was seized with a strange sense of general malaise, and called out for the nurse. In a few minutes he fainted; and, on reviving from this, he threw up some blood from his stomach, and expired.

On dissection, the stomach was found distended by an enormous clot of blood; but the most careful examination could not detect the source of the hæmorrhage.

Some of the sudden deaths which we hear of, and which are not investigated by dissection, are caused in this way. Was the above congestion in the vessels of the stomach a fatal substitute for the ague paroxysm that ought to have occurred?

---

30. Case of Catalepsy. By Mr. North.

Hysteria, the mocking-bird of nosology, appears far more frequently in the garb of other diseases than in its own common and unequivocal character. Catalepsy, we consider as one of the Proteian shapes of that multiform disease, in despite of the authority of Cullen, who looked upon it as a species of apoplexy, and, indeed, doubted its existence—of Frank, who regarded it in the light of a convulsion, and of Sauvages, who refers it to comatose debility. We think Mr. North is right in applying the term *Hysterical,* to the case of Catalepsy, which he has lately related in our cotemporary, the Medical and Physical Journal, and which we consider to be a fair specimen of that rare form of disease. The late Dr. Good, with his usual fondness for multiplying distinctions without difference, places catalepsy in the genus Carus, making it the second species, the first being Ecstasia. One of the best authenticated cases, of late years, is that published by Dr. Gooch, in the last volume of the College Transactions, and analyzed in a former number of this Journal. But we shall now notice Mr. North's case.

The patient, as usual, was a young female, who arrived in London, much fatigued by her journey, and in a state of great mental anxiety, resulting from a love-affair. First, she had pain and swelling in one of her feet—then intense pain in her head, with slight hysterical paroxysms, &c.—in short, each day presented a new form of disease, which at last so puzzled her medical attendant, (not Mr. North) that he thought the devil must be in the girl! A physician was consulted, and pronounced the complaint hysteria. When Mr. North was called, the young woman was supposed to be dying. She was apparently in a profound sleep, into which she had fallen after a violent attack of hysteria. No motion could be perceived in any part of the body—no pulse in any artery—scarcely any action of the heart—no respiration—pupils contracted—temperature of the body below par. She drew in a gentle but deep inspiration about every ten minutes. *A stimulating enema, and four drops of oil of croton on the tongue.* She continued in this state for twelve hours, when a slight hysterical paroxysm dissolved the spell. The next shape in which Proteus appeared, after an interval of a few days, was a violent trembling of the whole body, succeeded by a short sleep, and then an attack of hysteria. In a day or two afterwards, the real cataleptic phenomena were developed. "She resembled a figure of wax which might be moulded to any form. In whatever position she was put, she remained as immoveable as a statue, however aukward and fatiguing it might be. She was, for instance, placed in a sitting position, with her arms in a boxing attitude, and thus she remained till the caprice of the bye-standers put her into some other form. One eye was opened to its full extent, the other at the same moment closed. It remained fixed, and the pupil as perfectly contracted and immoveable as before. The globe of the eye appeared quite insensible to the touch, as did the other parts of the body." On two or three occasions, she was placed in a standing position, with her limbs in various attitudes, which would, with difficulty, have been assumed, even for a moment, by a person in health, and which could not have been so long supported by voluntary efforts. When the nervous influence was expended, the muscles suddenly relaxed, and, if standing, she would fall as if struck by a cannon ball. She continued in this cataleptic state, with intervals of various duration, for a fortnight, and then the malady assumed the form of chorea. After this, her breasts swelled and became very painful. She was sent to St. George's Hospital, where the same curious alternation of symptoms occurred. She is now in the country, and still suffers from violent attacks of hysteria.

Mr. North makes many judicious observations on those diseases which partake of the hysterical character, and which mislead young practitioners so much, especially by inducing them to make use of depletive measures, which generally aggravate the hysterical disposition, and multiply the puzzling forms of disease. In most forms of hysteria, however, the secretions are depraved, and the bowels torpid or irregular. This was the case in Mr. North's patient, and authorized purgation. But even this measure may be carried too far in hysterical females. We have seen it do much mischief.

---

31. Incisions in Erysipelas Phlegmonodes.

The late debates in the Medico-Chirurgical Society (continued for three nights in succession) on Mr. Lawrence's paper on Erysipelas, have excited considerable sensation; but we shall defer all remarks upon the subject till the paper is published in the forthcoming volume of the Society's Transactions, when we shall dedicate a long article to erysipelas. In the mean time, in order that every man may have his due in the merit of introducing an important remedy (incisions) into practice, we shall lay before our readers the following extract and case, which latter occurred at the Westminster Hospital more than four years ago, and in which the practice of Mr. Hutchison was successfully employed by Mr. Guthrie long before it was introduced by Mr. Lawrence at Bartholomew's. The case will, also, tend to shew Mr. Guthrie's views as to the cause of the bad symptoms in erysipelas phlegmonodes, and the reason why relief is so suddenly obtained by one or two long incisions carried through the skin and cellular membrane, but not through the fascia.

"This species of inflammation is usually the consequence of injuries; the skin assumes the erysipelatous tint, although it is in general something of a brighter colour. The part swells more rapidly, does not admit of the impression of the finger being made with the same facility as in either common erysipelas, or in the œdematous inflammation, and does not retain the mark in the same manner. There is clearly a thickening of the parts beneath the skin, which is also evidently on the stretch, is very tense, and therefore glistening. The pain is considerable; it is not, however, either, or the whole of these symptoms which attract particular attention, it is the rapid depression and derangement of the nervous system. The altered and subdued appearance of the patient from the previous day, his hurried manner, the quickness and irritable state of the pulse, the foulness of the tongue, heat of skin, and towards night a state of wandering or delirium, indicating the extent of irritation. If relief be not obtained, the swelling extends along the limb, the skin becomes of a darker colour, the erysipelas affecting it passes beyond, and is the precursor of the inflammation of the subcutaneous tissue; the distinction between them is well marked, and cannot be mistaken. The firmness of the part first affected has by this time yielded in some degree; its resistance or elastic feel is less evident, and it has obtained a springy fluctuating feel to the touch, which is peculiar, and which it has acquired before any matter has formed. On making an incision into the part at this period, the cellular tissue will be found to have changed its characteristic for a gelatinous appearance of a light leaden colour, which it obtains from the deposition of fluid into its cells, nearly in the act of being converted into pus. The septa composing the cells have not at this period lost their life, and the fluid does not at first exude, as it will be found to do a few hours later, when the matter deposited has become purulent. When this change has taken place, the patient is obviously in the greatest danger, and if the cause of irritation be not removed or alleviated, he will in many instances die under the most marked symptoms of irritative fever of a typhoid type. When the powers of the constitution are equal to sustain and resist this state of disease, relief is obtained by the sloughing of the skin, and the discharge of the matter beneath. The skin is however exceedingly tough, and before it yields and dies, the fascia beneath the cellular membrane is often destroyed, and the muscles are implicated and exposed. Mr. C. Hutchison thinks 'pus is seldom formed in the substance of the adipose part of the tela cellulosa exterior to the aponeurotic expansion, that is, between this membrane and the skin; its most common position is beneath these parts, and in immediate contact with the muscles.' This opinion does not accord however with my observation; the sloughing of the fascia, and the formation of matter beneath being most frequently caused by the continuance of the disease, and rarely occurring when the proper method of treatment has been adopted. Mr. Hutchison recommends several small incisions to be made, about an inch and a half in length, and from two to four inches apart, varied in number from four to eighteen, according to the extent of surface the disease is found to occupy. I have found one or more longer incisions answer equally well, and they appear in many instances to be preferable, giving more decided relief, as one incision can sometimes be made so as to be very little remarkable, whilst several smaller ones occasion more deformity. On making an incision at an early period, the leaden-coloured and slightly gelatinous appearance of the cellular membrane will be readily perceived, and the state of tension of the skin will be immediately estimated by the retraction of the edges of the wound, one of four inches in length separating two in width. Sometimes a considerable quantity of blood will flow from the divided surface, but this will in general be greater if the incision be carried through the fascia, which is seldom necessary at an early period of the disease. If the operation has been delayed until the springy fluctuating feel, communicated by this gelatinous state of the cellular membrane, be changed into the more marked feeling which is communicated to the foot when stepping on a bog or quagmire, the cellular membrane will have been destroyed, the skin will have been undermined, a part of it must be lost, in spite of the operation, which will only be in time to allay the constitutional symptoms, and thereby perhaps save the patient. I attribute these violent constitutional symptoms, not to the formation of matter, or the sloughing of the cellular membrane, but to the stretching and over-excitement of the skin when in a state of inflammation, caused by the swelling of the parts beneath; whence the relief obtained from the incisions. This opinion seems to be confirmed by the fact, that the constitutional symptoms subside, and the patient is placed in safety, although the incisions should not have been made until after the whole of the cellular membrane had passed into a sloughing state, and which process must be afterwards completed, and the parts separated, before the cure can be accomplished. The following case is so striking an instance of the efficacy of long incisions, and of their capability to remove the greatest constitutional irritation, that I do not consider it necessary to adduce more.

" Thomas Key, aged 40, a hard drinker, admitted into the Westminster Hospital, as an accident, on October 21, 1823, at night, and under my care, in consequence of falling and striking his left arm against a stool four days previously, which had given rise to erysipelatous inflammation. He was smartly purged with calomel and jalap, on his admission, which was followed up the next day by small doses of the antimonium tartarizatum and sulphate of magnesia, so as to cause both vomiting and purging. In the evening he lost twenty-five ounces of blood from the temporal artery. The arm was very much swelled, the skin of an erysipelatous redness, very tense, elastic, springy, and yielding a sensation of fluctuation, the inflammation being evidently deep-seated; pulse one hundred and twenty, strong, tongue dry and furred, great thirst, skin hot, is very restless, unruly, and wandering. After the bleeding he became quiet, a profuse perspiration broke out over the whole body; he appeared relieved and comparatively tranquil. Fomentation, and poultices were applied every three hours to the arm.

" On the 30th, his state not being improved, a consultation was held to determine on the propriety of making incisions into the inflamed part; but this was considered improper by the parties consulted, and saline medicines with small doses of tinct. opii were substituted.

" October 31. *Pulse one hundred and thirty*, he is weaker and more irritable, was delirious all night, and in a state of great restlessness, countenance sunk, skin dry and hot, tongue furred, and altogether in a state of extreme danger. The arm greatly swelled, of a darker colour, and giving to the touch a strong fluctuating boggy feel. I made two incisions forthwith into the fore arm; one on the back part eight inches in length, the other five inches long on the under edge in the line of the ulna down to the fascia, which was in part divided, and one vessel bled freely. There was not any matter beneath it, but a considerable quantity of serum and matter of a gelatinous appearance was discharged, mixed with venous blood, but no pus. The incisions did not give much pain.

"November 1. *Pulse ninety* and steady; tongue furred, but rather moist; heat of skin moderate; slept occasionally during the night, and was much quieter; says himself he had a good night. The arm is less swelled; the cellular membrane is evidently sloughing, and this state extends beyond the extremities of the incision on the back of the arm, which was therefore augmented to the extent of eleven inches. Ordered to continue the saline mixture, four grains of calomel, and four of the extract of colocynth, and the infus. of senna and salts to be given afterwards, and repeated until a due effect is produced.

"From this time he gradually recovered, the incisions were made, however, too late to prevent the loss of a considerable quantity of cellular membrane and skin.

"When a deep-seated erysipelatous inflammation takes place below the fascia of a limb, the whole extremity swells, it becomes firm, heavy, of a dull whitish colour; and is scarcely affected by the erysipelatous blush; is painful, and rapidly destroys the powers of life; the patient sinks unconscious of his danger, when he fancies himself relieved. The appearance of the part on dissection very much resembles that noticed page 97. It is a fatal termination by no means uncommon in persons of a bad habit, afflicted with erysipelatous and gangrenous inflammation or sloughing abscess in the neighbourhood of the rectum."—*On Gunshot Wounds, 2d Ed. p.* 105—11.

Messrs. Moulin and Guibert have lately published a paper and some cases illustrative of the nature and treatment of this dangerous species of erysipelas, in which they condemn the practice of incisions, as unnecessary and cruel, and rely on great numbers of leeches to the limb or part, with the various other items of the antiphlogistic plan. This was the treatment recommended by Messrs. Brodie, Travers, and several eminent surgeons, in opposition to Hutchison, Guthrie, Lawrence, and some others. It appears, however, by a case related in the paper, that the celebrated Boyer is inclined to the practice of incisions. Of this case we shall give a few particulars.

*Case.* Mr. R. aged 52 years, of strong constitution, was bled in the arm on the 6th May, 1824, quickly after which, the limb swelled and became very painful. Cold applications were employed, but the inflammation advanced, and great fever came on. Emollient poultices were applied for two days, during which the tumefaction and other symptoms increased. On the fourth day, the inflammatory tension had arrived at the greatest degree—the pain was intolerable—the fever ardent—the prostration extreme, although the pulse was full and hard. The wretched patient had never slept a moment for three nights. At this time, Baron Boyer was called in, viz. on the 10th May, and recommended incisions, "not only to take off the inflammatory *tension*, but, also, to give vent to the purulent matter which had probably formed." The friends of the patient, however, would not admit of the operation, and all the symptoms became exasperated, with the addition of delirium. On the 11th, another consultation was held, at which Baron Boyer, M. Fouquier, and M. Guibert, assisted. It was now determined, as incisions would not be submitted to, that a number of leeches should be applied to the inflamed and swelled member. But it was too late—the patient expired at eight o'clock the same evening!—*Bibliotheque Med. Sept.* 1827.

Several other cases are related—some to shew the fatal effects of the bark and wine system, formerly too generally employed—and some to illustrate the good effects of the antiphlogistic regimen, with repeated relays of leeches to the part. But, as we shall soon return to this subject, we shall say no more on the present occasion.

---

### 32. New Remedy for Asthma.

Whoever is conversant with this distressing complaint, knows with what eagerness the afflicted patients fly to the window for fresh air, when attacked with a paroxysm of asthma. They experience relief from this procedure, or they would not have recourse to it. Indeed, it appears that, in pure spasmodic or periodical asthma, there is a peculiar constriction of the air-cells and finer bronchial tubes, which prevents the ingress of the atmosphere, and threatens suffocation. Impressed with this idea, which is certainly a natural one, Dr. Chiarenti, an Italian physician, himself a victim to this disease, had recourse to the insufflation of atmospheric air, by means of a common pair of bellows, in his own person. He introduced the pipe of the instrument into his mouth, and, closing the nostrils, he pushed the air forcibly into his lungs, and with instant relief. He continued the operation for a considerable time, and still with the same good effects. By this remedy, he put a speedy period to the paroxysm. He then extended the trial to others, under similar circumstances, and with similar benefit. After a mass of experience with this remedy, he comes to the conclusion, that the artificial insufflation of atmospheric air is not only a means of putting a speedy termination to the paroxysm of asthma, but of radically curing the disease, if organic alterations have not taken place in the lungs.—*Journal de Progres.*

---

33. Ingestion of Hot Water in Gout.

In a former Number of this Journal, we alluded to a strange practice introduced by M. Cadet de Vaux, namely, the swallowing immense quantities of hot water for the cure of gout and rheumatism. Dr. Kruger has recently stated a case where this plan was tried by a patient, and the following were the effects.

M. Scholz, aged 47 years, began to have gout in May, 1821, which continued to recur, in slight paroxysms, till March, 1826, when they were rather severe. He now read M. Cadet's work on hot water, and determined to test the remedy. On the 20th March he began with a tumbler of water as hot as he could drink it, every 15 minutes. The first few glasses threw him into a perspiration, which continued till he took the 30th dose, when nausea and vomiting supervened. He persevered with the hot water till he completed the 38th glass, (each contained about seven ounces) when his head began to turn, and he was unable to take any more. Epileptiform convulsions succeeded—and all the superficial veins of the body were remarkably distended—respiration stertorous—pulse unaffected—perspiration copious. Some æther was first given, and then 20 grains of ipecacuan, which did not vomit; neither could the bowels be moved by lavements. The patient had no power over the sphincter ani or the bladder. Diffusible stimuli were next given. Vomiting took place four hours after the exhibition of the ipecacuan, and was succeeded by a paroxysm of convulsions that left the patient in a state of great exhaustion, and complete privation of intellectual powers. Another convulsive paroxysm was followed by a few hours sleep, from which he awoke with renewal of intellectual functions, but without any recollection of what had passed during the indisposition. The gout, which had been seated in the great toe at the commencement of the imbibition of warm water, had now disappeared, and did not return for that time. The patient, however, experienced great debility for a considerable time afterwards, requiring a long course of tonics.

Another case is related by Dr. Kruger—that of an apothecary who, having had some distressing dyspeptic symptoms, became affected with gout. In a paroxysm of this last disease, in which the whole body was affected; with heat in the stomach, palpitation of the heart, constipation, and jaundice, the patient had recourse to the Sangrado practice, being tired and disgusted with medicine. He swallowed 40 glasses of hot water, each containing 7 ounces. The consequence was, an immense distention of the body—congestion about the head, delirium, vomitings, copious perspirations, and urinary discharges, &c. by which his strength was greatly exhausted, and his life put in imminent danger. The gout, in this case, did not subside from the feet, and it was a long time before he was able to walk.

These effects of copious ingurgitation of hot watery fluids are curious and interesting to the physiologist and pathologist. It is evident that a considerable portion, at all events, of the water must have passed through the circulation, and the effects on the brain were nearly similar in both cases. In the latter case, the abundant perspiration and urinary secretion may have prevented the paroxysms of convulsions, which were so conspicuous in the first patient.—*Bibliotheque Med.*

---

34. Inflammation of the Neck of the Bladder, with *Strictures* on the Use of the Bougie.

[Hospice de Perfectionnement.]

We introduce the following case, with the view of putting young (and, we are sorry to say, some *old*) surgeons on their guard against the inconsiderate use of bougies, where there is great sensibility in the urethra and neck of the bladder.

*Case.* A man, about 50 years of age, was brought into the above-mentioned hospital, in Paris, who had had several attacks of gonorrhœa. A catheter was introduced, by way of exploration, to see what could be found, and this introduction produced most exquisite pain, as soon as the instrument reached the neck of the bladder, with constant and painful mictu-rition afterwards, the urine being highly charged with tough mucus. The patient evinced some febrile symptoms, and disorder of the digestive organs. Dr. Cloquet now wished to throw into the bladder emollient injections, by means of a hollow sound; but was obliged to relinquish this measure, on account of the exquisite sensibility (vive sensibilité) of the neck of the bladder. Diluents were then employed, with low diet, for eight days, at the end of which time, there being no amelioration of the symptoms, balsam of copaiba was exhibited in pretty large doses. Fever, with great irritation of the digestive organs, forced Dr. C. to abandon this medicine. But it was too late; for the fever assumed a low type, and the patient died on the 20th day after his entrance into the hospital!!

*Dissection.* The contents of the head and thorax were healthy. There were some spots of redness, and other signs of irritation in the mucous membrane of the intestinal canal. The urethra was in its normal state, as far as the membranous portion, but here the most unequivocal signs of inflammation, with thickening of the lining membrane, commenced, and were continued to some distance within the bladder, where the inflammation and thickening gradually disappeared. The prostate gland was enlarged, and contained a quantity of pus, which was infiltrated into its parenchymatous structure, not collected into a focus.—*Archives Generales.*

*Remarks.* We think it will hardly be denied, that this unfortunate patient met with an untimely end by the imprudent interference of the surgical measures that were pursued. Had this man been treated by leeches to the perineum and anus—barley water—and gentle aperients, with hyosciamus, instead of bougies and balsam copaiba, we have no doubt that he would have been living and well at the present time! We have frequent opportunities of witnessing the injurious effects of bougies in irritable states of the urethra and neck of the bladder; but the foregoing case happening *abroad*, is introduced as a hint to some of our chirurgicals at *home*.

---

35. Luxation of the Cervical Vertebræ.

[M. Pinault. Hôpital Cochin.]

James Bove, aged 31 years, fell on his head from a height of 20 feet, and was carried in a state of insensibility to the above-mentioned hospital, where he soon recovered his senses, and answered correctly to questions. All parts of the body below the arm-pits were completely paralytic. In the upper extremities, also, the sensibility and muscular power were considerably diminished. On examining the spinal column, a depression was observed opposite the fifth cervical vertebra. On pressing this part, the patient complained of acute pain. The respiration was carried on by the diaphragm alone, there being no motion of the ribs. Priapism. He was largely bled. On the second day, the patient was nearly in the same state, except that the upper extremities were completely paralyzed. The respiration was more and more difficult, and he died in a state of asphyxia in 24 hours more.

*Dissection.* The cervical ligament was found to be torn, and there was a complete luxation of the fifth from the sixth cervical vertebra, without any fracture. At this spot, the spinal marrow was compressed by the fifth vertebra. The lungs were gorged with blood. It does not appear that any means were used to reduce the luxation. Would any means have proved serviceable?

---

36. Case of Emphysema of the Substance of the Heart; with Observations on Valvular Disease of the same Organ.

A very curious case of this rare disease came lately under the care of Mr. Morrah, of Sloane Street, and was attended by that gentleman and Dr. Johnson. The patient was a captain in the Royal Navy, aged about 52 years, who had met with some misfortunes a few years ago, and afterwards began to evince symptoms of disordered action of the heart, namely, dyspnœa on going up stairs, and irregularity of the pulse. These symptoms gradually increased, till, at length, he was confined to his bed-room, though never to his bed. About four months ago, when examined carefully with the stethoscope, the heart was found to beat over a large space, and to be very tumultuous and irregular in its action. The whizzing sound was also very audible, when the ventricles contracted. The contraction could frequently be heard through the stethoscope, when no corresponding pulse was felt at the wrist. Thus, when not more than 50 or 60 strokes of the pulse could be enumerated, and those very irregular, there might be heard 70 or 80 contractions of the ventricle. The respiration was audible in all parts of the chest, except in the region of the heart, which occupied a large space, and consequently prevented the breathing being heard in that quarter. At this time, there were alternate paroxysms of dyspnœa and calm breathing, the intervals varying from a few minutes to as many hours. The night, however, was very distressing, as he could not lie low in bed, and his sleep was disturbed by startings and sense of suffocation. There was nothing very particular in the other functions. His dyspnœa was generally relieved by bleeding, local or general, with blisters, aperient medicine, and low diet.

The diagnosis was, enlargement of the heart, and imperfection of the valvular apparatus, without any material affection of the lungs. For two months before this unfortunate gentleman's death, his sufferings were very great, especially in the night. His time was passed in alternate paroxysms of dyspnœa, threatening every moment his life, and then sudden cessation of the attack, with an interval of *complete freedom from all dyspnœa.* It was very curious to observe that the interval (for more than a month prior to death) was almost invariably twice as long as the paroxysm. Thus, if the dyspnœa lasted five minutes, the patient had a complete immunity for ten minutes afterwards. The accessions were, like the cessations, *instantaneous.* He would be conversing quietly with his wife, his children, or his medical attendants, and in the very midst of a sentence, or even of a word, he would be seized with such a panting for breath, that a stranger would suppose he was in articulo-mortis. Equally sudden and abrupt would be the termination of this terrible conflict; and then he would take up the sentence or the word where it had been interrupted by the paroxysm.

Towards the close of the scene—that is, for the last three weeks of the patient's life, there were evident symptoms of effusion in the chest, as well as œdema of the lower extremities. Then the respiration could not be heard below the middle of the thorax, in the perpendicular position, though it was quite audible all round the superior parts of the chest. He suddenly expired while conversing with his daughter, in one of the intervals of dyspnœa, on the 23d of October, as he was sitting in his arm-chair. The body was examined on the 24th, by Mr. Morrah, Dr. Johnson, and Mr. Stevenson, of the Edgeware Road.

There was very considerable effusion, amounting to several pints, in the two sides of the chest. The heart was nearly three times its natural size—all its cavities being greatly dilated, but its parietes not being increased in proportion. The auricles were nearly of their natural thickness, though their cavities were considerably augmented. The right ventricle was passively enlarged. Its parietes were thinner than natural, though its cavity was more than double its proper size. The cavity of the left ventricle was also more than double its usual dimensions, but the parietes presented certain remarkable appearances. In some places, they were full an inch in thickness—in others, not a quarter of an inch. Near the apex of the left ventricle, the muscular structure was white, and condensed into a substance almost as firm as ligament. Between this and the basis of the ventricle, there were some portions of the walls not more than a fifth of an inch in thickness. The basis of the ventricle, on the contrary, presented parietes full an inch in thickness—but this thickened portion was every where emphysematous, and air could be pressed from it, with a crackling noise, exactly as from a piece of lung. By firm and continued pressure these thickened parietes could be reduced to less than half their dimensions, when the air was completely expelled.

The openings between the auricles and ventricles, on both sides, were greatly enlarged. The auriculo-ventricular opening, for instance, on the left side, was at least an inch and a half, or two inches, *in diameter*; and, as the mitral and tricuspid valves shewed no increase of dimensions, it was manifest that the functions of these valves were very imperfectly performed. When the left ventricle, for example, contracted, a great portion of the blood must have passed back into the auricle, from the inability of the mitral valve to cover the auriculo-ventricular opening. This would satisfactorily account for the pulse at the wrist not always being felt in correspondence with the ventricular contraction, as heard through the stethoscope. The same imperfection must have existed in the right side of the heart. The right ventricle, instead of throwing its blood completely into the pulmonary artery, ejected a portion of its contents back into the auricle. Thus the venous blood from the larger circulation was retarded, and eventually led to dropsical effusions; while the blood returning from the lungs to the left side of the heart must have experienced great interruption, and, consequently, the vessels of the lungs were kept in a state of congestion.

The emphysema of the parietes of the left ventricle cannot be accounted for on any known principle of the animal economy, but the fact is undeniable. The lungs themselves were crepitous, and sound throughout.

In respect to the tumultuous noise which was heard in this case, through the stethoscope, it resembled more the noise of CHURNING, than that which has been compared to the purring of a cat—the stroke of a saw—or the blast of a small pair of bellows, by Laennec and others. There can be little doubt that it was produced by the regurgitation of blood from the ventricles into the auricles, at each ventricular contraction, owing to the inability of the mitral and tricuspid valves to close completely their respective auriculo-ventricular openings. To render imperfect the office of the valves of the heart, it is not necessary that they themselves should be indurated, puckered, or otherwise changed in structure. If the opening which the valve is designed to cover be unnaturally dilated, the same effect will be produced as if the valve were ossified or contracted—and this is a pathological condition which is often overlooked. On examining very carefully those hearts which have been morbidly dilated in their cavities, whatever was the condition of the parietes, it appeared to the writer of this, that the valves were almost always incapable of effectually covering their respective apertures, and, therefore, that the circulation of the blood through the heart was imperfectly carried on. If the *right* chambers of the heart be much enlarged, and the valvular apparatus be consequently damaged, there will be no irregularity of the pulse, for that depends entirely on the *left* ventricle; but there must, in such a case, be great derangement of the general venous circulation, and the ultimate tendency to serous effusion will be consequently established, however regular the pulse may be at the wrist. If, in such cases, we could feel the pulmonary artery, we would find intermissions of pulsation there. When the left chambers are dilated, and the valvular function impaired, the venous system of the lungs must necessarily be kept in a loaded state, and hence the dyspnœa which invariably attends this pathological condition of the heart. Hence, too, the temporary relief which the breathing experiences from bleeding, and other kinds of depletion.

---

37. OPERATION FOR EMPYEMA REPEATED.

In Horn's Archives, for March, 1826, there is related the case of a man, who twice underwent paracentesis thoracis, with an interval of 22 years between the operations.

*Case.* A man, of middle age, was seized with symptoms of violent inflammation in the left side of the chest, for which he was copiously bled, and had proper medicines administered. When questioned by Dr. Guerard, he informed the physician that, 22 years previously, he had been affected with pneumonia, which terminated in an abscess, and for which an operation was performed. As the inflammation still continued, Dr. G. ordered farther depletion, blisters, &c. On the 5th day, there was oppression on the chest, and the stethoscope gave indications of a fluid in the left cavity of the pleura. On the 9th day, the symptoms were so unequivocal, that the operation was performed, when a large quantity of fetid pus was evacuated, with relief of all the symptoms. The discharge gradually decreased, and, on the 5th day from the operation, the patient was able to leave his bed. He was seen a year after this period, in perfect health.

---

### 38. Perforation of the Intestinum Ileum.

[M. Martinet. Hôtel Dieu.]

It was reserved for modern pathology to discover the true cause and the terrible consequences of this fatal accident. A knowledge of the *causes* of a disease, is the first and most certain step towards prevention. In former times, when dissections were less common, many sudden and violent deaths were caused by perforations of the stomach and bowels, which were not suspected—and when these perforations were discovered, their cause was not ascertained. This cause is preceding inflammation and ulceration of the mucous membrane in general—sometimes, though very rarely, of the peritoneal coat first, and the other coats afterwards. When we reflect on the frequency of these intestinal *ulcerations*, we might wonder that *perforations* are so rare; but we must remember, that the peritoneal covering of the intestinal canal presents a most powerful and obstinate barrier to the escape of fæcal matters, even when the mucous and muscular coats are completely destroyed. Nevertheless the perforation does sometimes occur, and that at all periods of muco-enteritis—and even during convalescence from that disease, and from fevers, when patients are thought to be out of danger, and when practitioners are off their guard. Excesses of the table, at this period, bring the danger into view when it is too late. On this account, the convalescence of a patient from any disease in which inflammation of the internal surface of the intestinal canal had previously existed, should be most rigidly watched, and all imprudences in food forbidden.

When this rupture of the external tunic takes place, there is, in general, a pain, more or less acute, felt in some particular point of the abdomen, commonly about the umbilicus, or towards the caput coli. This pain is suddenly developed, and sometimes consists, at the very beginning, of a sense of burning heat, spreading in all directions from a centre. The pain, however, rapidly augments, and soon occupies the whole of the abdomen, rendering the least pressure insupportable. Then come on nausea, vomitings, hiccup, profound alteration of the features, extreme smallness and quickness of the pulse; all shewing the existence of acute peritonitis. Two, three, or four days of intense suffering bring the solace of death.

The diagnosis of this terrible calamity is rendered very difficult, when the patient happens, at the time, to labour under cerebral disease, especially arachnitis; for then, the delirium which obtains prevents the patient from feeling, or at least expressing, the abdominal sufferings. In a few instances, the inflammation resulting from perforation and extravasation has been sub-acute, and patients have lived ten or twelve days. This, however, is rare. If the pain continues confined to a circumscribed spot, we may hope that the intestine has been ruptured into a kind of cyst, of which there are several examples on record. We shall now introduce the particulars of a case in illustration.

*Case*. Brisson, aged 25, had fever and diarrhœa for three days prior to his entrance into the Hôtel Dieu, when he complained of abdominal pains, head-ache, and febrile attacks, every evening. *Second day*. The belly was rather painful on pressure, especially about the navel —diarrhœa—cephalalgia—tongue very white—pulse strong and full. *Injection containing tartar emetic and sulphas magnesiæ*, which provoked some vomiting, with relief of the cephalalgia and abdominal pain. During the next few days, he was kept on farinaceous food, and he was on the point of full convalescence, when, on the eleventh after his entrance, he was suddenly seized with severe pain above the pubes, which quickly spread over the abdomen, attended with dysury, vomitings, bloody diarrhœa, &c. *Leeches, warm-bath*:—but no relief followed. He lingered out for three days after this, and then died.

*Dissection.* The cavity of the pelvis was filled with flocculent pus and a milky fluid—intestines covered with false membranes, of very recent formation—a small perforation, perfectly circular, in the ileum, a few inches from the ileo-cœcal valve—the mucous membrane around the perforation was red, but not thickened, or otherwise altered—no other ulceration in the ileum; but there were two or three small ulcers in the large intestine, with inflammation of some of the mucous follicles.—*Revue Med.*

Happily, perforations, where there are so few ulcerations as in the above case, are very rare; and we find that Nature is more disposed to heal numerous and extensive ulcers in the mucous membrane, than to permit a perforation of the serous coat. Still we ought to be on our guard, and keep patients on mild nutriment, till all danger is completely over.

---

### 39. Gangrene of the Lungs.

This is a rare disease, and has never been accurately described before Laennec's time. Since the work of this talented physician appeared, M. Andral, M. Bouillaud, and others, have brought forward examples of the disease in question. The following case is deserving of record, though we shall greatly abridge the original.

*Case.* A gendarme, 38 years of age, received, on the 10th March, a violent blow of the fist on the right side of the chest, which was followed by severe pain for some days, but not such as to prevent him from pursuing his usual avocations. Towards the end of March, he began to lose his appetite, and decline in health. On the 16th April, he was forced to give up work, and consulted Dr. Pichot, who found the skin dry and hot, pulse quick and hard, tongue red at the point and edges, epigastrium tender on pressure, nausea, sometimes sickness after food, bowels irregular. The patient did not now complain of the side that had been injured; but only observed that he felt some uneasiness there one or two hours after eating. Considering the disease as inflammation of the mucous membrane of the stomach and bowels, Dr. P. ordered leeches to the epigastrium, and mucilaginous and farinaceous drinks. The patient was speedily relieved, and in ten days he was able to take food without inconvenience. Returning, however, to irregularity and intemperance, he fell back, in three weeks, to the same condition as before. The original treatment was again prescribed, and in three or four weeks he again recovered. Once more he got into his usual habits of intemperance, and again he relapsed. He now entered the Charenton Hospital, whence he was discharged uncured. He next tried the Val de Grace, and had no better success. He returned home, and consulted a Parisian physician, who promised to set him up in a fortnight. Emetics and drastic purgatives were now energetically administered, and the patient daily got worse! On the 15th September, Dr. P. was again consulted, and found the poor man in a desperate condition, being emaciated to a skeleton, and harrassed with constant expuition from the mouth, without cough, or difficulty of breathing. The patient complained of burning pain between the shoulders and in the right side of the chest—pulse soft and regular—urine high-coloured—sleep greatly interrupted—abdomen swelled and painful in several places. Our author suspected chronic gastro-enteritis, converted into acute or sub-acute; but did not suppose there was any very material disease of the lungs. He prescribed some soothing medicine, and, for a few days, the patient seemed a little better. At the end of a week, he was suddenly seized with a sense of suffocation, extreme pain in his back and through the chest, violent cough, attended with fetid expectoration. These symptoms were sometimes exasperated—sometimes moderated; and in this condition the miserable patient dragged out a wretched existence for fifteen days, when he expired.

*Dissection.* In the right side of the thorax, there was found a vast depôt of purulent matter, enclosed in a kind of cyst, formed by false membranes organized. The greater part of the lung of that side was in a state of complete gangrene, being black, putrid, soft, and exhaling a most fetid odour. The left lung was the seat of a considerable degree of inflammation, as was the internal surface of the pericardium, which was covered with a soft false membrane. The heart itself was sound. The liver was greatly enlarged, and somewhat indurated. The stomach and intestines were opened throughout their whole extent, and various marks of chronic inflammation were evident on their internal surface.—*Bibliotheque Medicale.*

We conclude, from the particulars of the above case, that chronic inflammation and disorganization had been going on in the right lung ever since the time the man had received the injury there, and that acute inflammation having supervened about the 20th September, it ran into gangrene, and thus produced this rare specimen of disease in the pulmonary structure. The case affords an instructive lesson respecting the insidious nature of pulmonic inflammation sometimes, and may serve as a caution against looking too much to the abdominal organs, while mischief is going forward in the chest. This oversight is daily committed in this country.

---

### 40. Signs of the Times, or Epidemics of the Day.

A few years ago, there arose, all at once, an epidemic rage for PHLEBOTOMY. An experiment was made on a plethoric member of the church, who was bled by the founder of this epidemic system, *usque ad deliquium*, for several weeks in succession—and that for the cure of a disease not to be found in the Nosologies of Cullen or Mason Good—but which has been termed by the phrenologists a PROPENSITY. This experiment excited a great sensation, both in the medical and clerical professions; but, for some reason or other, the church, which could have so well afforded to be bled profusely, was suddenly abandoned, and the founder of the system turned his LANCET against the more conspicuous bodies of his own brethren, and bled at so furious a rate, that a universal atrophy was apprehended by the heads of the profession! Sir Astley Cooper, from the stamina of his constitution, and the plenitude of his vascular system, was selected as the first *medical* patient of the phlebotomist. He was bled daily—not by ounces, but by pounds—and this extraordinary depletion was carried on for more than a year! To the surprise of every one, the vigorous baronet bore this daily and hebdomadal venesection and *sanguisuction* (for *leeches* were also employed in great numbers) without apparent exhaustion. But every thing has its limits, and a period at last arrived, when even Sir ASTLEY COOPER would bleed no more!

Mr. ABERNETHY was known to be very far gone in a certain *derangement* of the stomach and bowels, which blue pill and black draught entirely failed to remove. Indeed, his disorder was said to be on the increase, and the PHLEBOTOMIST thought him an excellent *patient* for a trial of the new remedy. Without the formality of a *consultation*—indeed, without leave or licence, he thrust his LANCET into the body of the lecturer, and *abstracted* the vital fluid at a tremendous rate, affirming that he would soon *correct*, not only the morbid secretions of Mr. A.'s chylopoietic viscera, but that he would condense certain gazeous exhalations from his pineal gland, for the sole good of the public—and not with any view to his own private emolument. The eccentric patient did not prove quite so tractable as Sir Astley Cooper. He winced and kicked most lustily, and protested that neither his stomach nor his head required or desired any such phlebotomising measures. Blood-letting did not suit his constitution—and he would take nothing but his own favourite pill. These remonstrances made no impression on the *phlebotomist*. On the contrary, he very deliberately proceeded to the operation of the TREPAN, boring the cranium day after day, and extracting a portion of his patient's brains, which he exposed for sale, in the Strand, with as much *sang froid*, as a butcher would sell the brains of a sheep in Leadenhall Market!!

This was "too bad," as Lord Liverpool once said; and, therefore, Mr. Abernethy applied to the Lord Chancellor (who has the especial care of men's cerebral property) for an injunction against the skull-boring and brain-sucking operations of a surgeon, whose professional advice or assistance he never solicited. Here was a puzzling question for Lord Eldon! Mercy on us! what a world of consideration was necessary to determine so knotty a point! What important rights and privileges might be invaded or compromised, if one surgeon were restrained from perforating the skull and extracting the brains of another, whether invited to operate, or doing so by way of experiment! Long did the Chancellor weigh these mighty matters in his deepest thoughts—but, by the time he had "made up his mind" on the subject, the poor patient's head was as full of holes as a sieve, and his cranium as empty as that of Yorick!

It has been said of some animals, that, after tasting human flesh, they could never relish any thing else. It was so with the PHLEBOTOMIST. The brains of Abernethy were so delicious and so *fattening* withal, that the brains of Clutterbuck, Bell, Lawrence, Armstrong, Haslam, Blundell, Brande, &c. were hashed up, and so well *cooked* by a COMPANY of PHLEBOTOMISTS, which was now *established by charter*, that public entertainments were weekly given (at 2, P.M. every Friday) to all who chose to partake of the *intellectual* banquet, at the very *ordinary* price of eight-pence a *head*.

Now we do not find fault with the company for these their public treats. On the contrary, we believe that the individuals above enumerated, had each a great deal more brains than were necessary—and that, as a certain number of the profession are by no means overburthened with these articles, so a kind of public distribution, for the preservation of an equilibrium, was very charitable, and had all the good effects of the largesses which used to be occasionally scattered among the hungry Romans, when it was thought politic to put them in good humour with their masters.

But men will, in time, get tired of the most delicate viands; and a faint murmur of "*toujours perdrix*," was a strong hint for varying the bill of fare. The PHLEBOTOMISTS had excellent noses for smelling out savoury matters; and the hospitals, the dissecting-rooms, the dispensaries, and even the colleges of the land, furnished a most odoriferous variety of garbal for garnishing out the dishes of *brains* at the *head of the table*. All this, too, had its use; and we are far from censuring the diligence of the Worshipful Company of Phlebotomists, in thus uniting the salutary trade of public scavenger with that of public cook. The rooks are always respected as useful birds, on account of their propensities to pick up worms and insects. Even the unsightly ADJUTANT is permitted to strut through the streets of Calcutta unmolested, because he devours every putrefying and deleterious substance that comes in his way. But there is no unmixed good in this world. Whether it was that a dearth of "morbid parts" did sometimes occur, or that the *company's* sense of smell did sometimes deceive them, but so it was, that living, healthy, and useful *limbs* of the profession came to be "served out," and served up, at almost every banquet—till, at length, a general dread of *mutilation* began to pervade the more corpulent and portly members of medical society—while the stomachs of the guests, and even of the bye-standers, shewed symptoms of *turning* at sight of the horrible spectacle of human sacrifices before them. The offences of this COMPANY were at last rank, and "smelled to Heaven;" for, like Diana of old, in the Island of Taurica, they had their INSTRUMENT—a very sharp one, too—as an Iphigenia, ready to immolate every one who did not belong to their association!

A strong re-action was at last visible in the public mind; and a sense of common danger, prompted to measures of common defence. A lucky thought occurred to some one, that an ANATOMICAL COMPANY might be formed, whose office it would be, to *dissect* the "morbid parts" of the old or PHLEBOTOMIST COMPANY, and form a museum of these precious specimens, to be exhibited in conspicuous parts of town and country.

It has been currently reported, but for the accuracy of the report we will not vouch, that this lucky thought of an Anatomical Company, occurred at a festive meeting of the committee of manslaughter; and that, while the juice of the Tuscan grape was flowing round in healths, "five fathom deep," the *vice-chairman* was sharpening his scalpel for a dissection of the *president*.

It is a remarkable fact, however, and may serve as a lesson to short-sighted mortals, that, on the *very day* when it was triumphantly proclaimed, "that opposition and competition were at an end," the new joint-stock company started into existence, to the utter dismay and discomfiture of the original *monopolists*.

But, whatever may have been the *origin* of the Dissecting Company, it is certain that the appearance of this *opposition* to the phlebotomists excited a very strong sensation. Several very neat dissections and specimens of "morbid parts" were quickly forwarded to the new company; and men began to wonder why they had so long permitted an association of scandel-bearers to revel on their vitals, without retaliating in kind on their persecutors. The workings of the public mind were not unheeded by the Old Company of Phlebotomists—and the first measure which suggested itself to the Directors was, to lower the price of their ordinary —or rather to place a few *supplemental* covers on their table, containing various kinds of most *suspicious* viands, in order to induce their guests to continue customers. This was a doubtful expedient. It was like offering some *additional* messes of fat pork to a company of sea-sick passengers. In short, the medical profession now began to perceive, for the first time, that they had been taking great pains to place themselves in a *false position*, with regard to the public at large, and especially with regard to the other learned and liberal professions. It was no longer to be concealed, that the public felt disposed to question the *taste* of the medical faculty, and to compare them with a community of *cannibals*, who preyed on each other, in open day, without the least sense of sin or shame! The consequences of this revelry on human flesh were now unequivocal. The manners of the medical profession had *retrograded* four centuries, in the short space of four years! On searching the records of their art, no specimens of defamation could be discovered, that might at all compete with those exhibited by the Company of Phlebotomists. It was evident that some years of *reform* and repentance would hardly be sufficient to set the medical profession on a level with their neighbours, in point of polished manners and *refined taste*, for which they had hitherto obtained credit. The work of reformation was a Herculean task. The Augean stable was to be cleansed, and people were some time in determining on the stream that was to wash away the filth.*

---

* We beg it may be distinctly understood that we do not identify the *ostensible* editor, or rather the Proprietor of the Lancet, with the real editors or writers in that publication. We have the best reasons for believing that Mr. Wakley is not the writer of those scurrilous, defamatory, and odious articles, which appear, and have so often appeared in that periodical. We believe they were and are penned by hireling editors, who, in betraying the confidence of their master, have injured the medical profession at large. The following portraits of two ex-editors, drawn in the very last number of the Lancet, will explain our meaning.

"We are told, however, that Mr. "Guthrie intends to rely chiefly on the "testimony of two individuals, whom we "do not hesitate to pronounce the most "ungrateful, and the most short-sighted "*scoundrels* that ever disgraced their, or "any other profession—men, whose mo-"ral *turpitude* has gone *immeasurably* "beyond the ordinary course of *human* "*crimes*. Give us the opportunity, kind, "modest author, of publicly extracting "from these wretches *a history of their* "*own infamy*, and we will ever after call "thee friend."—*Lancet, 22d December*, 1827.

For the credit of human nature, we hope that such monsters, whoever they be, have not existed, even as the hireling writers of the Lancet! The above document, however, shews in what light Mr. Wakley holds those, whose bold, manly, and independent writings have captivated a certain portion of medical society!! In helping to expose the delinquencies of such hirelings, Mr. Wakley will be guilty of the blackest ingratitude, if he does not hereafter call us—friends!

We have the very best means of knowing that the great majority of the profession abhor the CALIBANISM of the instrument in question, and only suffered themselves to be polluted by its pages for the sake of useful information commixed with abominations of all kinds—and the exposure of abuses which, unfortunately, have existed, and still exist. But, as hurricanes purify the air, and as political convulsions and the ebullitions of vicious passions call forth the best and most virtuous energies of the human mind, so the company alluded to has been the instrument of good as well as evil. It has called forth a phalanx in the cause of honour, virtue, talent, liberality, science, and truth, which will redeem the character of the English medical profession, and shed a lustre on human nature itself. We are no observers of the signs of the times—we know nothing of the springs that actuate men's mind's, or we are right in prognosticating that the year 1828 will exhibit a display of talent, knowledge, and high-minded probity, which will drive into the abyss of despair, if not of oblivion, the host of MALIGNERS of the medical character, which have so long revelled in the abuse of a power which they vainly thought to be absolute, unlimited, and unassailable.

Whether or not we have uniformly, and hitherto *almost singly* resisted this diabolical tyranny, it is for our readers to bear witness. We have now numerous allies in the work of stemming the torrent of demoralization; but the most powerful of all, is, without doubt, the reaction of the public mind itself. This is so evident to the JUNTO, that some of its members, with that instinctive sagacity which characterizes a very small quadruped—the RAT, are backing out of the company, with all possible haste. The *point* of their instrument indeed is broken off—or rather it is completely worn out by its own venesectionary mal-practices—and those who used to shrink in alarm, when the weapon was unsheathed, now begin to laugh it to scorn.

In fine, as we now hope and believe that the taste for defamation and vulgar abuse is rapidly declining, so the claims for public favour will turn on, and be decided by, the superiority of talent, extent of information, and judicious industry of the competitors. Under such a system, the more numerous the candidates, the greater will be the exertion; and the human intellect will now be called upon for the display of its utmost energies. An enemy to all monopolies, we rejoice at the prospect which expands before the eye of philanthropy. In the intellectual contest, which has now spread to the medical profession, we should grieve at the extinction of any rival, however formidable and however inimical. In drawing the sword against every thing which tends to degrade the profession to which we are enthusiastically attached, we hold out the olive branch to every honorable competitor in the promotion of medical science and medical literature.

---

### 41. Phlebitis.

[Dr. Forbes. Med. Chir. Trans. Vol. XIII. Part II.]

The second part of volume thirteen of the above transactions has recently appeared, and a volume is now to be published regularly every six months. A year or two ago, the LANCET, that oracle of truth, proclaimed to the world that the Medico-Chirurgical Society was on its death-bed. This oracular prediction is fulfilled, in the way that all its other prognostications are verified—as, for example, the pupillary insurrection at Bartholomew's Hospital. A considerable number of members and visitors are obliged to go away frequently from the meetings of the Society, for want of room, and the list of its members is rapidly increasing. So much for the moral influence of the JUNTO'S JOURNAL.

The present volume or part contains a great variety of articles, of unequal merit, and interest, no doubt, but still calculated to sustain the character and celebrity of the Society. We shall take care to give a complete analysis of all the more valuable and practical papers, in as quick succession as possible, though in the present instance we can do little more than announce the publication of the volume, and the flourishing state of the Society.

CASE OF PHLEBITIS.

This case was read at the Society on the 27th February, 1827, and the morbid preparation exhibited to the members. It was considered by those who have adopted Dr. Davis's doctrine of phlegmatia dolens as an excellent corroboration of that doctrine; but our readers will soon see the weakness of the support thus obtained.

The patient was a young man of 26 years, conceived to be labouring under pulmonary consumption, accompanied by "swelling of both ankles." The swelling of the right ankle, however, gradually subsided, while that of the left extended upwards and increased, attended with pain about the ankle and calf of the leg, knee, ham, groin, and lower part of the abdomen. With these symptoms, the whole limb became hotter than the other, and tender on pressure. Dr. Forbes saw the patient for the first time on the 31st October, a week before his death. He was then in the last stage of pulmonary consumption. The limb was double the size of the other, "and every where it pitted on pressure." The colour of the skin was whiter than natural, and presented the exact resemblance of "common anasarca." The subcutaneous veins above the ankle were distinctly marked, and distended with blood. Died on the 8th November.

The dissection was performed by Mr. Grainger. The saphena major and its branches were greatly distended, and filled with coagulated blood, but their coats were not diseased. The cellular tissue of the whole limb was infiltrated with a limpid fluid, and the lymphatic glands in the groin were rather enlarged. The femoral vein was filled with coagulated blood.

"The dissection was continued so as to expose the left iliac veins. The trunk of the external and common iliac vessels was found even more distended than the femoral vein. The common iliac vein had an unnatural colour, approaching to a greenish hue; and it seemed as large as the inferior cava. The distension of this vessel suddenly terminated at that part where it is united with the right common iliac vein. The left internal iliac vein was filled with blood for about two inches of its course, and the rest of it appeared natural.

"The femoral and iliac veins on the right side were nearly empty, and quite healthy in their appearance, forming a strong contrast, when compared with the gorged and distended state of the veins on the left side.

"On laying open the upper part of the femoral vein, as well as the external and common iliac veins, they were found filled with a coagulum, of much firmer consistence than is usually met with in healthy veins; and had much of the fibrous appearance of the blood which is found contained in aneurismal sacs. On separating this coagulum, a thin but distinct membranous layer was seen adherent to the internal coat of the vein, and it required some force to separate them. The femoral vein, which was examined as far down the limb as the triceps muscle, was found in the same condition." 296.

We perfectly agree with Dr. Forbes that the morbid appearances in this case were very similar to those which have been described by Dr. Davis in his paper on phlegmatia dolens—but on that very account we maintain that this was not a case of phlegmatia dolens, but one of phlebitis. In a former number we have taken such pains to disprove Dr. Davis's theory, that we need not recur to the subject again. If phlegmatia dolens depended on inflammation of the inguinal and iliac veins, three-fourths of the patients would die—whereas death does not take place in one case in the hundred where that disease is exquisitely marked. This fact would be sufficient to disprove the identity—but there are numerous other reasons for refusing acquiescence in Dr. Davis's views.

---

42. Extra-uterine Pregnancy.

Mr. Norman, of Bath, has related an interesting case of this kind, in the last vol. of the Med. Chir. Transactions—and the more so, on account of its termination. Eight months before the date of report, the female, aged 41, had become very ill, with palpitation, difficulty of breathing, frequent sickness and vomiting. It was then the catamenia were interrupted. At three months from this cessation, a rounded tumour was felt in the left iliac region, about the size of a fist. She could not lie on her right side, and all her distressing symptoms were on the increase. At five months from last menstruation, she felt the motions of the child, which gradually became stronger, until all doubt of pregnancy was at an end. Her breathing became more difficult, and she was often unable to lie down at night. At the end of eight months, (14th October) Mr. N. was summoned. She had been suddenly seized with symptoms indicative of failure in the action of the heart, and her dissolution seemed impending. Large doses of opium restored the heart's action, and a few days passed before any thing was done. On the 13th October, she was again seized with a paroxysm, and again she was relieved by laudanum, &c. It was determined to induce labour, as she was now supposed to be in the ninth month; but, on examination, the os uteri could not be discovered. The form of the child's head was distinctly felt, covered by an intervening substance, as thick as the parietes of the uterus. Through this an incision was made, about two inches in extent; but still the os uteri could not be found. Some fluid escaped, and the scalp of the child's head could be felt. She became easier, and remained so through the night and next day. In the evening, she had another paroxysm of dyspnœa, on recovering from which, it was agreed to open the child's head, as it was far out of the reach of the forceps or vectis. The head was opened, and the brain extracted, as was afterwards the body of the child. No placenta presented, or could be felt. A hard solid tumour was found on the left side of the abdomen. It was now considered that the case was one of extra-uterine conception, and that the opening had been made through the vagina and peritoneum. On the second day, symptoms of peritoneal inflammation came on, and she soon died.

On dissection, the small intestines were found much inflamed, as well as the peritoneum. The placenta was found attached to the right ligamentum latum—the funis was torn away about two inches from the placenta—the part divided in the operation was discovered to be the posterior portion of the vagina—the os uteri was situated above the pubes, as in retroversio uteri. The uterus itself was thickened and hard, and its cavity was lined with a well-defined tunica decidua. The heart was flaccid—the ventricles thin, and containing no blood. Mr. Norman thinks that, could the placenta have been extracted, and had there not been disease of the uterus, the recovery of the patient was not improbable. It appears to us, that the patient had disease of the heart, (passive aneurism) which would have soon terminated her existence.

---

### 43. Duration of Pregnancy.

In a paper in the recently-published volume of the Medico-Chirurgical Transactions, Dr. Merriman has given some tables calculated to assist in throwing light on the mean duration of utero-gestation. We say the mean duration, for we have no doubt that Nature, in this, as in many other instances, deviates occasionally, and to a considerable extent, from her own general laws.

Of the births of 114 mature children, calculated from, but not including, the day on which the catamenia were last distinguishable, 3 happened in the 37th week—13 in the 38th week—14 in the 39th week—33 in the 40th week—22 in the 41st week—15 in the 42d week—10 in the 43d week—and 4 in the 44th week.

From this table, our author thinks it fair to infer, that conception is effected soon after the catamenial period has intermitted, rather than immediately before the expected return of that period. A knowledge of this fact, he says, is useful on many occasions, and is of paramount importance, by enabling the accoucheur to fix upon the proper time for inducing premature labour in cases of deformed pelvis. Our experienced author has seen a very few cases where the period of delivery (calculated from the last appearance of the catamenia) exceeded 44 weeks, or 308 days; and of these few anomalous cases, he has given some details in the paper above-mentioned.

